# Deletion of the African swine fever virus E120R gene completely attenuates its virulence by enhancing host innate immunity and impairing virus release

**DOI:** 10.1080/22221751.2025.2555722

**Published:** 2025-09-03

**Authors:** Boli Ma, Yiqian Jiang, Nan Li, Fengjie Wang, Qian Li, Huixian Yue, Yanyan Zhang, Rongliang Hu, Faming Miao

**Affiliations:** a College of Life Sciences, Ningxia University, Yinchuan, People’s Republic of China; b State Key Laboratory of Pathogen and Biosecurity, Key Laboratory of Prevention & Control for African Swine Fever and Other Major Pig Diseases, Ministry of Agriculture and Rural Affairs, Changchun Veterinary Research Institute, Chinese Academy of Agricultural Sciences, Changchun, People’s Republic of China

**Keywords:** African swine fever, African swine fever virus, E120R, immune evasion, virulence factors, protective efficacy

## Abstract

African swine fever virus (ASFV) causes a lethal haemorrhagic disease in domestic pigs and poses a major threat to the global swine industry. Currently, no effective commercial vaccines or antiviral drugs are available for ASF control. In this study, we constructed a recombinant E120R gene-deleted virus, ASFV-ΔE120R, based on the highly virulent genotype II strain SY18, to investigate the role of the E120R gene. ASFV-ΔE120R exhibited impaired virion release and formed aberrant tubular structures, rendering viral particles more susceptible to neutralization by convalescent pig sera. ASFV-ΔE120R induced higher levels of transcription of Cytokines, chemokines, and interferon-regulated genes in porcine alveolar macrophages compared with ASFV-WT. In vivo safety evaluation demonstrated that piglets immunized with 5 × 10⁶ TCID₅₀ of ASFV-ΔE120R exhibited no clinical signs or viral nucleic acid in tissues at necropsy on days 4, 7, 10, and 14 post-immunization. Two immunizations at the same dose, 21 days apart, also induced no clinical signs or viral shedding during a 28-day observation. Immunogenicity analysis showed that ASFV-ΔE120R elicited p54-specific antibodies and IFN-γ-secreting PBMC responses. Upon challenge with parental ASFV SY18, two of five pigs (40%) survived, showing elevated antibody levels, IFN-γ-secreting PBMCs, and increased CD8^+^ IFN-γ^+^ T cells. Moreover, Cytokines and interferon-stimulated genes were significantly upregulated in survivors. In summary, ASFV-ΔE120R is fully attenuated, safe, and induces both humoral and cellular immune responses, highlighting pE120R as a rational target for ASF vaccine development.

## Introduction

African swine fever (ASF) is a highly lethal viral disease of pigs, with a case fatality rate approaching 100%  [1]. The disease has spread to numerous pig-producing countries, causing substantial economic losses and posing a serious threat to global food security. In efforts to control ASF outbreaks, significant research has been devoted to vaccine development worldwide. Rationally designed live attenuated vaccines (LAVs), generated by targeted deletion of ASFV virulence-associated genes, have demonstrated promising immunogenicity and protective efficacy under experimental conditions [2,3]. However, two critical challenges hinder the clinical application of such vaccine strains: reversion to virulence and viral shedding. Previous studies reported that the commercially released ASFV-G-ΔI177L vaccine strain may regain virulence after serial passage in pigs, with third- and fourth-generation vaccinated animals developing typical ASF-specific clinical signs [4]. In addition, most reported gene-deleted vaccine strains exhibit varying degrees of viral shedding [3,5–7]. If residual attenuated virus in the environment recombines with other ASFV genotypes, reversion to virulence may occur. For example, Dongming Zhao et al. found that recombinant ASFV strains of genotype I/II exhibited high lethality and transmissibility, and that LAVs derived from genotype II ASFV failed to confer protection against these recombinant viruses [8]. Therefore, ensuring both protective efficacy and environmental biosafety, particularly through the prevention of post-vaccination viral shedding, is a critical prerequisite for the practical deployment of ASFV LAVs.

ASFV belongs to the family *Asfarviridae* and is a large double-stranded DNA virus that replicates in the cytoplasm of host cells  [9]. Its genome encodes approximately 150–167 proteins, many of which remain functionally uncharacterized  [10]. ASFV primarily targets the mononuclear phagocyte system in pigs, especially alveolar macrophages. Its multilayered virion structure – comprising the genome core, core shell, inner membrane, icosahedral capsid, and outer envelope – plays an essential role in virus entry, replication, assembly, and egress  [11,12]. Outer membrane proteins such as pEP402R are involved in viral entry; while pEP402R is not essential for infection, it is required for virus binding to red blood cells [13]. The capsid is primarily composed of pB438L, p72, and pE120R, and the inner membrane contains proteins such as p17, pE183L, and pE248R involved in assembly  [14]. Notably, pE120R has been reported to interact with the major capsid protein p72 and localize to the surface of intracellular virions, although it was not identified as a structural capsid component in high-resolution cryo-EM analysis [11,15]. Formation of the core region depends on proteolytic processing of the polyprotein precursors pp220 and pp62 by the viral protease pS273R  [16,17]. pE120R is a late-expressed structural protein of approximately 120 amino acids. Although previous studies suggested that it plays dual roles in viral trafficking and host immune modulation  [15,18–20], E120R has also been reported to be essential for virus replication, with repeated failures in generating recombinant ASFV strains lacking the E120R gene [18,19]. To date, no systematic cell-based or animal-based studies investigating E120R deletion in the context of highly virulent field strains have been reported, and whether E120R can serve as a rational “low-shedding, safe vaccine target” remains to be elucidated.

In this study, we constructed an E120R-deleted ASFV mutant (ASFV-ΔE120R) through homologous recombination and obtained a genetically stable virus via limiting dilution. Compared with the parental ASFV SY18 strain, ASFV-ΔE120R exhibited defective virion egress and enhanced activation of host antiviral responses *in vitro* the mutant was completely attenuated and exhibited no detectable viral shedding, while conferring partial protection against challenge with the parental virulent strain *in vivo*. Collectively, our findings demonstrate that ASFV-ΔE120R is both safe and immunogenic, and represents a promising candidate for the development of a live attenuated ASF vaccine.

## Results

### Construction and in vitro biological characterization of the ASFV-ΔE120R Deletion mutant

**Figure 1.**
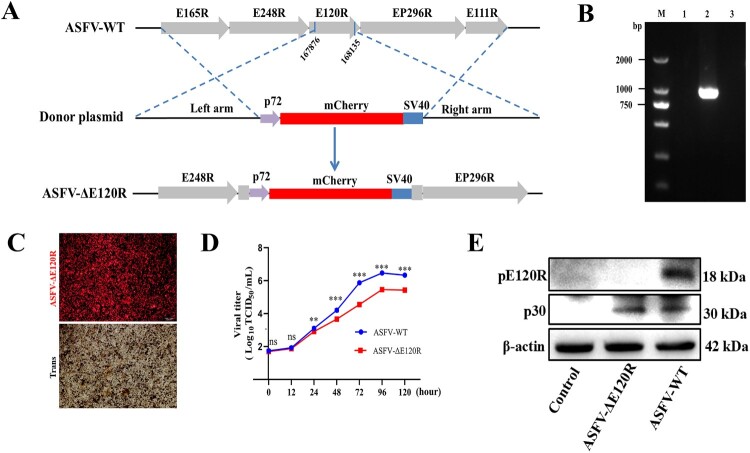
Purification and in vitro replication characterization of ASFV-ΔE120R. (A) Schematic of the homologous recombination strategy for constructing the E120R-deleted virus. Experimental details are provided in the Materials and Methods section. (B) Agarose gel electrophoresis of PCR products for purity verification of ASFV-ΔE120R. Lanes 1–3 show amplification results using E120R-specific primers on DNA extracted from ASFV-ΔE120R-infected cells, ASFV-WT-infected cells, and uninfected cells, respectively. (C) PAMs infected with ASFV-ΔE120R. (D) PAMs were infected with ASFV-WT or ASFV-ΔE120R at an MOI of 0.01, and viral titres were measured from total cell cultures (i.e. combined supernatant and cell pellet) at 0, 24, 48, 72, 96, and 120 hpi. (E) Western blot analysis of p30 and pE120R expression in PAMs infected with ASFV-WT or ASFV-ΔE120R at an multiplicity of infection (MOI) of 1. Cells were harvested at 24 hpi, lysed, and probed with specific antibodies. β-actin served as the loading control. Data represent three independent experiments. ASFV-WT vs ASFV-ΔE120R, **P < 0.01; ***P < 0.001; ns, not significant, *P* > 0.05.

**Figure 2.**
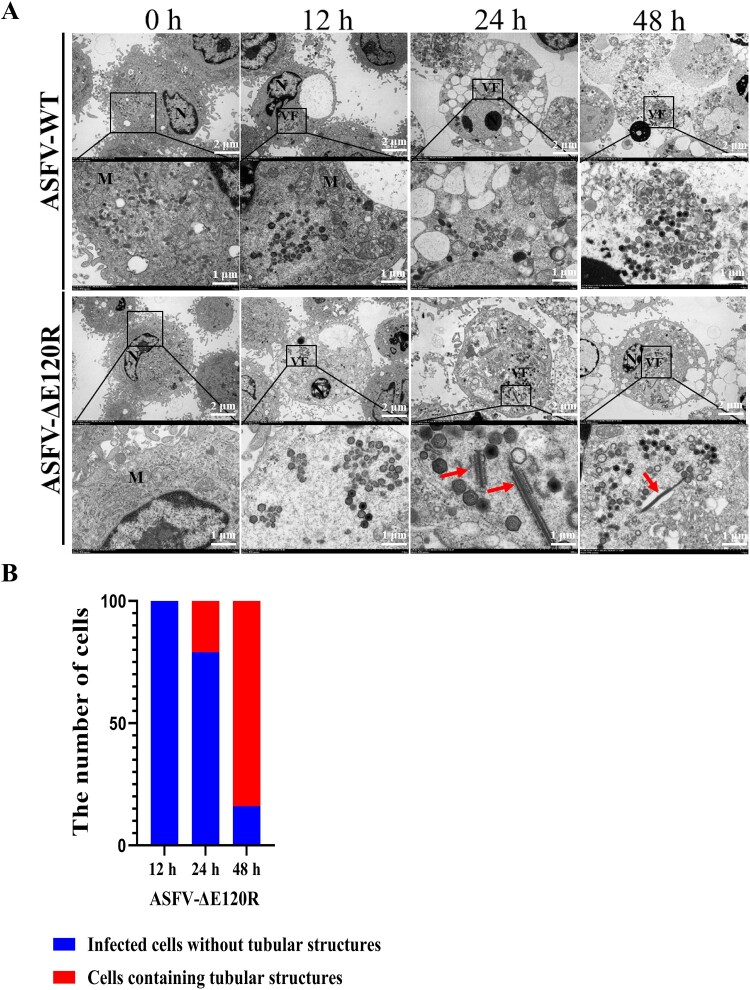
Virion morphology of ASFV-WT and ASFV-ΔE120R. (A)Morphological analysis of ASFV-WT and ASFV-ΔE120R virions. PAMs infected with ASFV-WT or ASFV-ΔE120R (MOI = 1) were harvested at 12, 24, and 48 hpi. Viral particles were visualized by TEM. N: nucleus; M: mitochondria; VF: viral factory; red arrows: tubular structures. Scale bar = 2 μm. (B) Quantification of tubular structures in ASFV-ΔE120R-infected cells. A total of 100 intact infected-cells were randomly counted for each group (ASFV-ΔE120R and ASFV-WT) at 12, 24, and 48 hpi. Representative images from 5 to 10 cells per sample were selected and included as supplementary material.

This study generated a recombinant ASFV (ASFV-ΔE120R) with deletion of the *E120R* gene from the highly pathogenic ASFV SY18 strain (ASFV-WT) through homologous recombination. The *E120R* gene was replaced by a reporter cassette containing the ASFV p72 gene promoter and mCherry (Figure 1(A)). The recombinant virus ASFV-ΔE120R was isolated by limited dilution method. To assess the purity of the recombinant virus, viral DNA extracted from ASFV-ΔE120R or ASFV-WT was subjected to PCR amplification using specific primers targeting the E120R gene. The results showed detectable amplification of the E120R gene in DNA from ASFV-WT, but not from ASFV-ΔE120R (Figures 1(B,C)), indicating successful purification of the ASFV-ΔE120R recombinant virus. Whole-genome sequencing analysis of ASFV further confirmed that the *E120R* gene at positions 167,876–168,135 of ASFV SY18 was replaced by the mCherry cassette. Additionally, genomic variations were identified, including an AC insertion at position 430 (non-coding region), a CCC deletion at position 13,275 (MGF110-14L, Gly113-Tyr112), a GGG deletion at position 19,041 (non-coding region), a GG insertion at position 20,843 (non-coding region), and an A insertion at position 47,376 (A104R, Lys6) (Table S1). Western blot analysis demonstrated no detectable pE120R expression in ASFV-ΔE120R, whereas pE120R was detected in ASFV-WT (Figure 1(E)). To investigate the impact of *E120R* on ASFV replication in vitro, porcine alveolar macrophages (PAMs) were infected with either wild-type ASFV (ASFV-WT) or recombinant ASFV-ΔE120R, and viral titres in the total cell culture (i.e. combined supernatant and cell pellet) were measured post-infection to generate growth curves. The results showed that from 24 to 120 h post-infection (hpi), deletion of the E120R gene significantly reduced the replication level of ASFV-ΔE120R in PAMs compared with ASFV-WT (Figure 1(D)). Transmission electron microscopy (TEM) was subsequently employed to examine the morphology of ASFV-ΔE120R and ASFV-WT virions. The results showed that ASFV-ΔE120R was capable of forming viral factories and producing icosahedral virions similar to those of ASFV-WT. However, starting at 24 hpi, abnormal tubular structures began to appear in cells infected with ASFV-ΔE120R. To further assess their frequency, 100 infected cells were randomly selected for each group (ASFV-WT and ASFV-ΔE120R) at 12, 24, and 48 hpi. No tubular structures were observed in ASFV-ΔE120R-infected cells at 12 hpi. At 24 hpi, tubular structures were detected in 21 out of 100 infected cells (21%), and the proportion increased markedly to 84% (84/100) at 48 hpi. In contrast, such structures were not observed in any of the 100 cells examined in the ASFV-WT group at all three time points (Figure 2(A,B)). Collectively, these findings confirm the successful purification of ASFV-ΔE120R, which exhibits significantly attenuated in vitro replication capacity compared to ASFV-WT and generates anomalous tubular structures during virion morphogenesis.

### ASFV-ΔE120R shows reduced release of infectious particles into the supernatant

**Figure 3.**
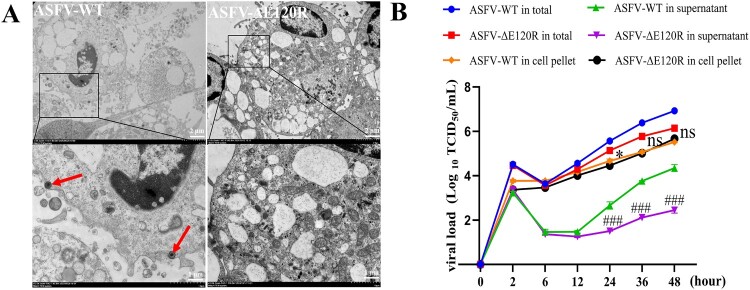
Impaired virion release in ASFV-ΔE120R-infected cells. (A) PAMs infected with ASFV-WT or ASFV-ΔE120R at an MOI of 1 were harvested at 24 hpi. Virion morphology was analysed by TEM. Red arrows: budding virions; Other supplementary electron microscope images are all displayed in the supplementary materials. (B) PAMs were infected with ASFV-WT or ASFV-ΔE120R at an MOI of 1. After 2 h of incubation, the inoculum was removed, and cells were washed three times with PBS. Cell pellet, supernatants, and in total (pellet and supernatants) were then collected at 2, 6, 12, 24, 36, and 48 hpi to quantify viral titres by TCID_50_ assay. Data represent three independent experiments. ASFV-WT in cell pellet *vs* ASFV-ΔE120R in cell pellet, *P < 0.05, ns, not significant; ASFV-WT in supernatants *vs* ASFV-ΔE120R in supernatants, ^###^*P* < 0.001.

To investigate the in vitro replication characteristics of ASFV-ΔE120R, PAMs were infected with ASFV (MOI = 1) and harvested at 24 hpi for transmission electron microscopy (TEM) analysis of virion morphology. We randomly examined 250 ASFV-WT- or ASFV-ΔE120R-infected PAMs by TEM. In the ASFV-WT group, budding virions were observed in 5 cells, each containing 1–2 budding ASFV particles, and no prominent budding structures comparable to those reported by Andrés et al. were captured [15], Additional images capturing viral budding from cells have been provided in the supplementary materials. In contrast, among 250 ASFV-ΔE120R-infected cells at 24 hpi, no budding virions were detected, and even virions near the plasma membrane were rarely observed (Figure 3(A)). To further determine whether deletion of the E120R gene affects virion release, PAMs were infected with either ASFV-WT or ASFV-ΔE120R at an MOI of 1. Supernatants, cell pellets, and whole-culture mixtures (supernatant + pellet) were collected at 2, 6, 12, 24, 36, and 48 hpi and subjected to TCID₅₀ assays. At 24–48 hpi, the virus titres in supernatants from the ASFV-WT group were significantly higher than those from the ΔE120R group by 1–2 log₁₀ TCID₅₀/mL. In the corresponding cell pellets, ASFV-WT showed similar titres to ASFV-ΔE120R, with differences of approximately 0.2 log₁₀ TCID₅₀/mL (Figure 3(B)). These findings indicate that deletion of the E120R gene substantially impairs the release of infectious virions.

### Serum from ASFV-convalescent swine inhibits ASFV-ΔE120R replication in PAMs

**Figure 4.**
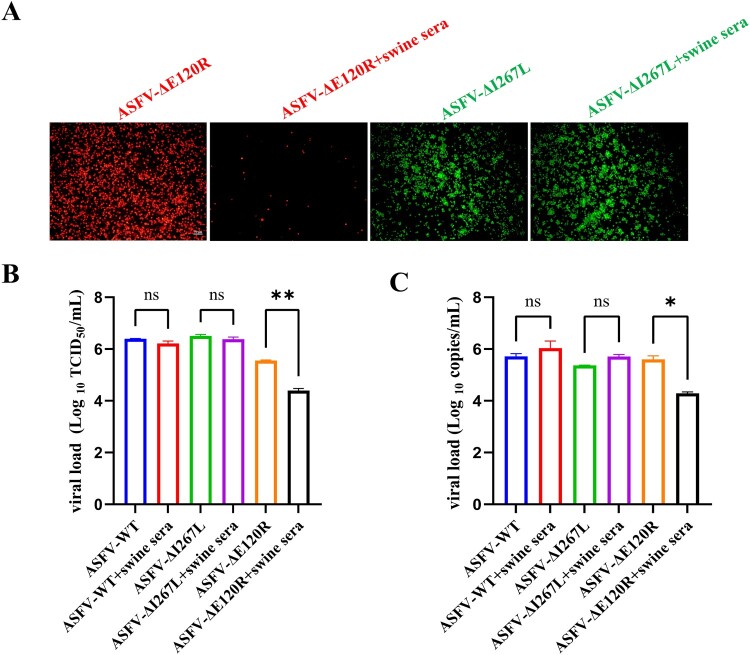
Serum neutralization assay of ASFV-WT, ASFV-ΔI267L, and ASFV-ΔE120R. (A) ASFV-WT, ASFV-ΔI267L, or ASFV-ΔE120R was pre-incubated with serum from ASFV-convalescent swine (the serum was diluted 1:10 using RPMI 1640) at 37°C for 2 h, followed by inoculation into 96-well plates containing PAMs. After 144 h of culture, fluorescence-positive cells were quantified by fluorescence microscopy and imaged. (B-C) Viral titres and genome copy in infected PAMs were determined by TCID_50_ assay and qPCR. Data represent three independent experiments. *P < 0.05; **P < 0.01; ns, not significant, *P* > 0.05.

To further investigate the immunogenic characteristics of ASFV-ΔE120R virions, serum from ASFV-convalescent swine (the serum was diluted 1:10 using RPMI 1640 medium) was pre-incubated with ASFV-WT, ASFV-ΔI267L, or ASFV-ΔE120R for 2 h, followed by inoculation of PAMs and incubation for 144 h. Viral titres and genome copies in cell cultures were subsequently quantified. Fluorescence microscopy revealed significant inhibition of ASFV-ΔE120R replication by the convalescent serum but not ASFV-ΔI267L (Figure 4(A)). Further analysis by TCID_50_ assay and qPCR demonstrated that pre-incubation with convalescent serum significantly reduced viral titres and genome copies of ASFV-ΔE120R compared to untreated controls. In contrast, the serum exhibited no significant inhibitory effects on ASFV-WT or ASFV-ΔI267L (Figure 4(B,C)). Collectively, these findings indicate that a subset of ASFV-ΔE120R virions can be neutralized by antibodies present in the convalescent serum.

### ASFV-ΔE120R induces stronger activation of antiviral immune signalling pathways and elevated production of interferon (IFN)-associated factors in PAMs compared to ASFV-WT

**Figure 5.**
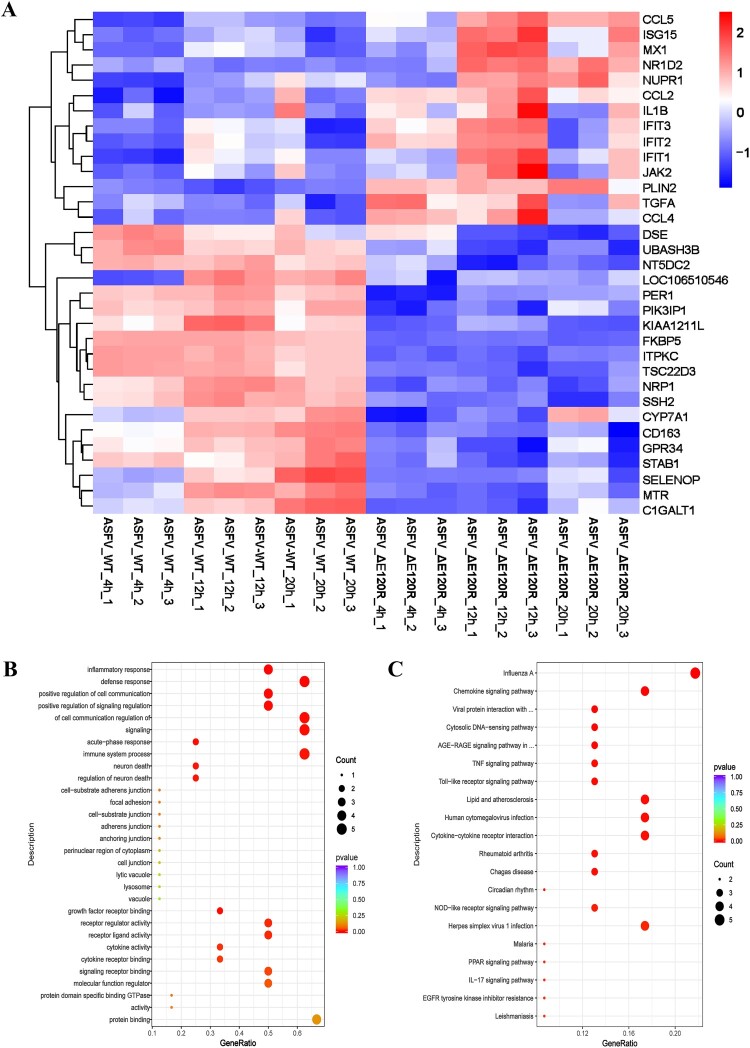
RNA-seq of ASFV-ΔE120R- and ASFV-WT-infected PAMs. (A) Heatmap of differentially expressed genes (DEGs) between ASFV-ΔE120R- and ASFV-WT-infected PAMs. PAMs were infected with ASFV-ΔE120R or ASFV-WT (MOI = 1) for 4, 12, and 20 hpi, followed by RNA-seq analysis. (B-C) Bioinformatic analysis of DEGs. GO enrichment (B) and KEGG pathway analysis (C) were performed on DEGs identified at 12 hpi between ASFV-ΔE120R and ASFV-WT-infected PAMs.

To investigate whether ASFV-ΔE120R activates host antiviral immunity in vitro, RNA-seq was performed to analyse antiviral immune responses in PAMs infected with ASFV-ΔE120R or ASFV-WT. Compared to ASFV-WT-infected PAMs, ASFV-ΔE120R induced significantly higher expression of chemokines (*CCL5*, *CCL2*, *CCL4*), interferon-stimulated genes (*ISG15*, *MX1*, *IFIT1-3*), and the inflammatory cytokine （*IL-1β*）, which are associated with inflammatory signalling and host antiviral immunity (Figure 5(A)). Gene Ontology (GO) and Kyoto Encyclopedia of Genes and Genomes (KEGG) enrichment analyses revealed that these genes were predominantly enriched in pathways related to innate immunity, inflammatory signalling, and host antiviral responses (Figure 5(B,C)). To further validate the enhanced induction of IFN-associated antiviral factors by ASFV-ΔE120R, RT-qPCR was conducted to measure mRNA levels of *IFN-α*, *IRF7*, *IRF8*, *ISG15*, *ISG54*, *ISG56*, *TNF-α*, and *MX1*. ASFV-ΔE120R significantly upregulated the expression of *IFN-α*, *IRF7*, *IRF8*, *ISG15*, *ISG54*, *ISG56*, and *MX1* at 12 and 20 hpi compared to ASFV-WT (Figure S1A-H). Collectively, these findings demonstrate that ASFV-ΔE120R activates innate immunity, inflammatory signalling, and host antiviral pathways in PAMs.

### ASFV-ΔE120R exhibits attenuated in vivo replication with no tissue pathology or viral shedding in immunized swine

**Figure 6.**
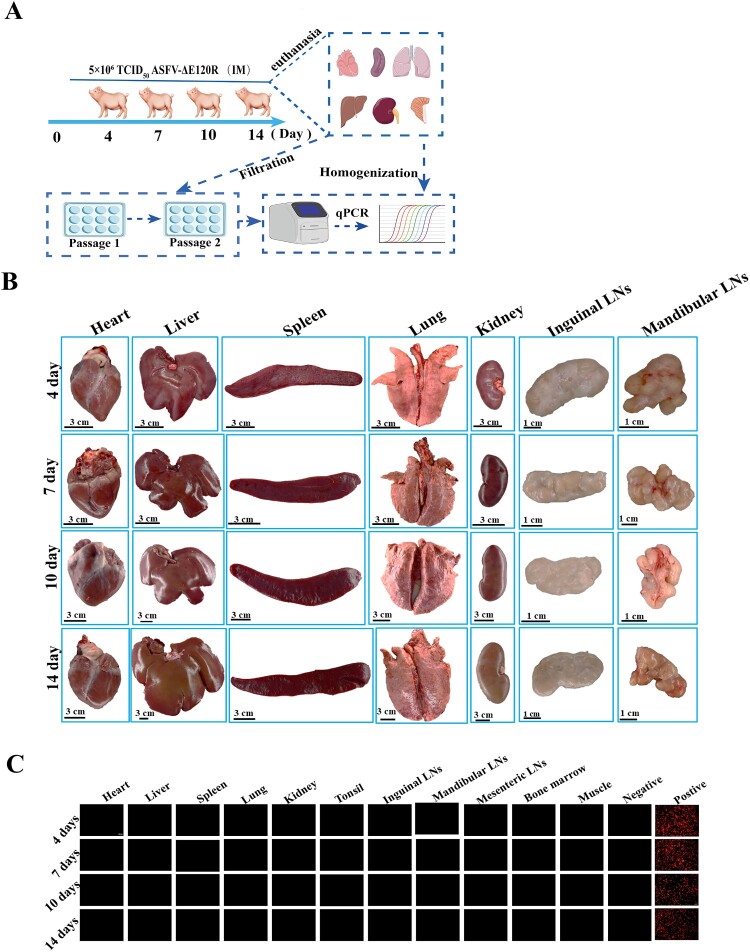
Analysis of tissue alterations and viral genome detection in pigs immunized with ASFV-ΔE120R at 4, 7, 10, and 14 days post-immunization. (A) Safety assessment design for ASFV-ΔE120R-immunized pigs. Twelve pigs were immunized with ASFV-ΔE120R (5 × 10^6^ TCID_50_), and three pigs per group were euthanized at 4, 7, 10, and 14 dpi. Tissues (heart, liver, spleen, lung, kidney, tonsil, mandibular lymph node, inguinal lymph node, mesenteric lymph node, bone marrow, muscle) were collected in duplicate for viral genome quantification by qPCR. Additionally, homogenized filtrates from heart, liver, spleen, lung, kidney, tonsil, mandibular lymph node, inguinal lymph node, mesenteric lymph node, bone marrow, muscle were inoculated into bone marrow-derived macrophages (BMDMs) to monitor number of fluorescence-positive cells. (B) Representative images of the heart, liver, spleen, lung, kidney, mandibular lymph node, and inguinal lymph node from ASFV-ΔE120R-immunized pigs at 4, 7, 10, and 14 dpi. (C) Number of fluorescence-positive cells analysis in BMDM cultures inoculated with tissue homogenate filtrates. Homogenates from heart, liver, spleen, lung, kidney, tonsil, mandibular lymph node, inguinal lymph node, mesenteric lymph node, bone marrow, and muscle of ASFV-ΔE120R-immunized pigs collected at 4, 7, 10, and 14 dpi were passaged twice in BMDMs (5 days per passage). Number of fluorescence-positive cells were assessed at the fifth day of the second passage.

To investigate the in vivo replication characteristics of ASFV-ΔE120R, twelve pigs were immunized with 5 × 10^6^ TCID_50_ of ASFV-ΔE120R, and three pigs per group were euthanized at 4, 7, 10, and 14 days post-immunization (dpi) for tissue collection (heart, liver, spleen, lung, kidney, tonsil, mandibular lymph node, inguinal lymph node, mesenteric lymph node, bone marrow, muscle). Viral genome copies in these tissues were quantified by qPCR, while tissue homogenates were filtered, inoculated into bone marrow-derived macrophages (BMDMs), and passaged twice (5 days per passage) to monitor number of fluorescence-positive cells and viral genome levels (Figure 6(A)). Tissue examination revealed no ASF-associated lesions in the heart, liver, spleen, lung, kidney, mandibular lymph node, or inguinal lymph node at any timepoint (Figure 6(B)). qPCR analysis detected no viral DNA in the heart, liver, spleen, lung, kidney, tonsil, mandibular lymph node, inguinal lymph node, mesenteric lymph node, bone marrow, or muscle of ASFV-ΔE120R-immunized pigs (Tables S2). BMDM cultures inoculated with tissue filtrates exhibited no fluorescence-positive cells, even after two passages, and subsequent qPCR of cell lysates confirmed that no viral DNA was detected (Figure 6(C), Tables S3). These results demonstrate that ASFV-ΔE120R caused no tissue pathology and exhibited undetectable viral shedding in immunized swine.

### ASFV-ΔE120R has certain immunogenicity and good safety

**Figure 7.**
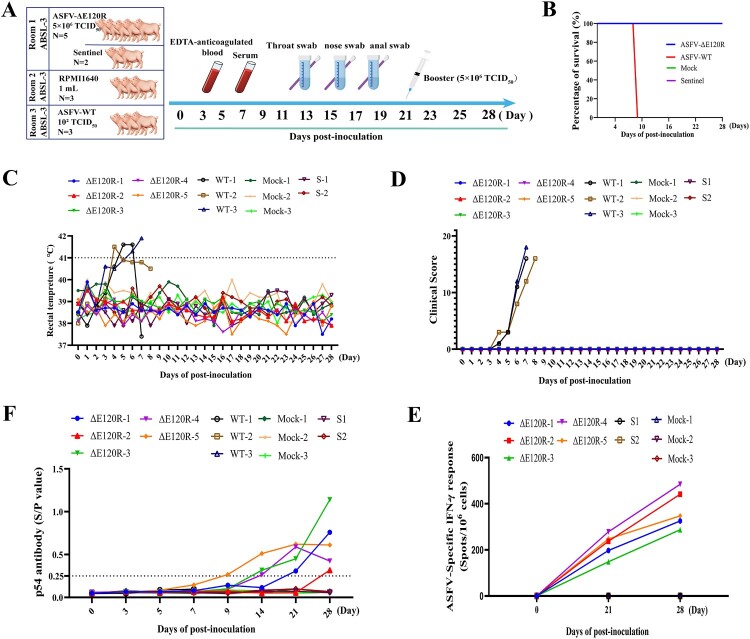
Immunization trial of pigs with ASFV-ΔE120R. (A) Experimental design: Pigs were intramuscularly (i.m.) immunized with ASFV-ΔE120R (n = 5, 5 × 10^6^ TCID_50_), ASFV-WT (n = 3, 10^2^ TCID_50_), or 1 mL RPMI 1640 (n = 3, Mock group). At 21 days post-primary immunization, ASFV-ΔE120R-immunized pigs received a booster dose (5 × 10^6^ TCID_50_, i.m.). Serum, blood, nasal swabs, oropharyngeal swabs, and anal swabs were collected at 0, 3, 5, 7, 9, 11, 13, 15, 17, 19, 21, 23, 25, and 28 days post-immunization. Survival rate (B) and rectal temperature (C) were monitored across groups. (D) Clinical scores of ASFV-ΔE120R, ASFV-WT, Sentinel (contact-exposed), and Mock groups over 28 days. (E) Anti-p54 antibody levels in ASFV-ΔE120R, ASFV-WT, Mock, and Sentinel groups measured by ELISA. (F) IFN-γ-secreting PBMCs in PBMCs of ASFV-ΔE120R, sentinel, and Mock groups at 21 and 28 days post-immunization quantified via ELISpot and expressed as mean spot-forming cells (SFC) per 10^6^ cells.

To evaluate the immunogenicity of ASFV-ΔE120R, pigs were immunized intramuscularly (i.m.) with 5 × 10^6^ TCID_50_ of ASFV-ΔE120R, 10^2^ TCID_50_ of the virulent ASFV SY18 strain (ASFV-WT), or 1 mL RPMI 1640 (Mock group). At 21 days post-primary immunization, pigs were boosted with ASFV-ΔE120R (5 × 10^6^ TCID_50_, i.m.) and monitored for 28 days (Figure 7(A)). Following ASFV SY18 challenge, all three pigs in the ASFV-WT group developed hyperthermia (>40.5°C) starting at 3 days post-infection, which persisted for 3–4 days, and succumbed to infection between 7 and 9 days post-infection. In contrast, pigs in the ASFV-ΔE120R, Mock, and Sentinel (contact-exposed) groups exhibited no clinical signs of ASF and maintained normal body temperatures throughout the 28-day observation period (Figure 7(C)). The ASFV-WT group showed 100% mortality, whereas all pigs in the ASFV-ΔE120R, Mock, and Sentinel groups survived (Figure 7(B)). Viral DNA was detected in blood, oropharyngeal swabs, nasal swabs, and anal swabs of ASFV-WT-infected pigs from 3 days post-infection until death at 9 days post-infection, whereas no viral DNA was detected in these samples from ASFV-ΔE120R, Mock, and Sentinel groups during the 28-day monitoring (Figure S2A-D). Serum samples collected during primary and booster immunizations were analysed for anti-p54 antibodies using an in-house ELISA. On day 21 post-immunization, three pigs in the ASFV-ΔE120R group (ΔE120R-1、ΔE120R-3, ΔE120R-4, and ΔE120R-5) tested antibody-positive (S/*P* > 0.25), while ΔE120R-2 showed S/*P* values of 0.05. Therefore, a booster immunization was administered. By day 28 post-immunization, all pigs in the ASFV-ΔE120R group were antibody-positive, with S/*P* values ranging from 0.31 to 1.14, while antibody levels in ASFV-WT, Mock, and Sentinel groups remained negative (Figure 7(F)). Peripheral blood mononuclear cells (PBMCs) from ASFV-ΔE120R, Mock, and Sentinel groups were assessed for IFN-γ secreting PBMCs. ASFV-ΔE120R-immunized pigs exhibited variable numbers of IFN-γ secreting PBMCs over 28 days (287-484 SFC/10⁶ cells), whereas no IFN-γ secreting PBMCs were detected in Sentinel or Mock groups (Figure 7(E)). Collectively, ASFV-ΔE120R demonstrated favourable safety and partial immunogenicity in swine.

### ASFV-ΔE120R has a certain ability to protect piglets challenged by parental strain

**Figure 8.**
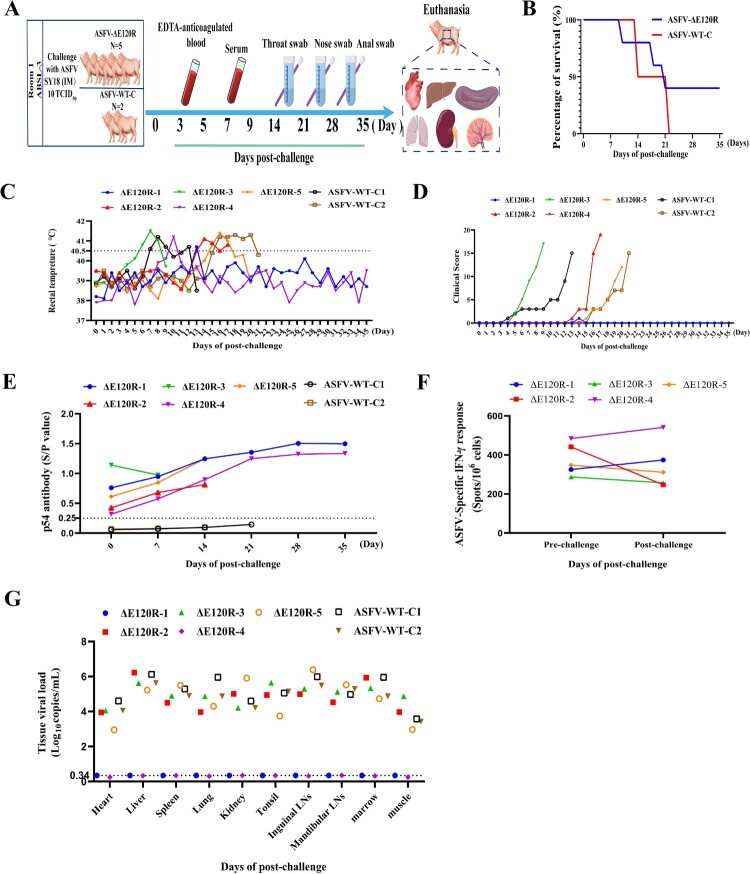
Challenge trial of ASFV-ΔE120R-immunized pigs. (A) Schematic of the viral challenge experiment. Pigs in the ASFV-ΔE120R group (n = 5) and ASFV-WT-C group (n = 2) were intramuscularly inoculated with ASFV SY18 (10 TCID_50_). Serum, blood, nasal swabs, oropharyngeal swabs, and anal swabs were collected at 0, 3, 5, 7, 9, 14, 21, 28, and 35 dpc. Survival rate (B) and rectal temperature (C) were monitored across groups. (D) Clinical scores of ASFV-ΔE120R (n = 5) and ASFV-WT-C (n = 2) groups following ASFV SY18 challenge (10 TCID_50_). (E) Anti-p54 antibody levels in ASFV-ΔE120R and ASFV-WT-C groups during the 35-day challenge period, analysed by ELISA. (F) IFN-γ-secreting PBMCs in PBMCs of ASFV-ΔE120R groups at pre-challenge and post-challenge quantified via ELISpot and expressed as mean SFC per 10^6^ cells. (G) Viral DNA detection in tissues of the surviving ASFV-ΔE120R-immunized pigs and ASFV-WT-C pigs. Surviving immunized pigs were euthanized at 35 dpc, and tissues (heart, liver, lung, kidney, spleen, tonsil, mandibular lymph node, inguinal lymph node, bone marrow, muscle) were collected for qPCR analysis. For pigs that died prior to 35 dpc, the same tissues were collected immediately post-mortem.

To assess the protective efficacy of the ASFV-ΔE120R strain against challenge with the parental ASFV SY18 strain, both ASFV-ΔE120R-immunized and ASFV-WT-C group pigs were infected with ASFV SY18 (10 TCID_50_) (Figure 8(A)). All pigs in the ASFV-WT-C group exhibited elevated rectal temperatures (>40.5°C) from 7 to 20 days post-challenge (dpc), accompanied by anorexia, lethargy, and other African swine fever (ASF)-associated clinical signs, which progressively worsened, resulting in 100% mortality within 21 days (Figure 8(B)). In the immunized group (ASFV-ΔE120R), ΔE120R-2, ΔE120R-3, and ΔE120R-5 pigs developed transient hyperthermia (>40.5°C) between 6 and 20 dpc, followed by severe ASF-related symptoms (persistent fever >40.5°C, anorexia, paralysis, convulsions), culminating in mortality by 20 dpc (Figure 8(B–D)). ΔE120R-1 and ΔE120R-4 pigs survived the 35-day observation period, ΔE120R-1 displaying a transient low-grade fever (40.5°C) at 13 dpc that resolved spontaneously (Figure 8(B–D)). High levels of viral DNA were detected in the blood, oropharyngeal swabs, nasal swabs, and anal swabs of pigs in the ASFV-WT-C group and three pigs in the ASFV-ΔE120R immunization group (ΔE120R-2, ΔE120R-3, and ΔE120R-5) from the period of infection with the parental virus until death. In contrast, during the 35-day observation period, no viral DNA levels exceeding those of the ASFV-WT-C group were observed in blood, oropharyngeal swabs, nasal swabs, or anal swabs of the two surviving pigs in the immunization group (Figure S3A-D). Anti-p54 antibody levels in serum samples collected during the challenge phase from ASFV-ΔE120R and ASFV-WT-C group pigs were analysed using an in-house ELISA. ΔE120R-1 and ΔE120R-4 exhibited a gradual increase in antibody titres during the first 21 days post-challenge, reaching S/*P* values of 1.35 and 1.24, respectively, followed by a plateau thereafter. In contrast, no significant changes in antibody titres were observed in pig ASFV-WT-C-1, ASFV-WT-C-2, ΔE120R-2, ΔE120R-3, ΔE120R-5 (Figure 8(E)). PBMCs from ASFV-ΔE120R groups were assessed for IFN-γ secreting cells. Within 35 days, ΔE120R-1 and ΔE120R-4 showed a certain level of increase in PBMCs that produced IFN-γ after challenge, which were 374 and 541 SFC/10⁶ cells, respectively. However, the PBMCs that produce IFN-γ in ΔE120R-2, ΔE120R-3 and ΔE120R-5 did not increase (Figure 8(F)). At the end of the 35-day observation period of ASFV SY18 challenge, heart, liver, spleen, lung, kidney, tonsils, mandibular lymph nodes, inguinal lymph nodes, bone marrow, and muscle tissues were collected from pigs in the ASFV-WT-C group and two live pigs in the ASFV-ΔE120R immunization group for viral DNA detection. A relatively high level of viral DNA was detected in the pig tissues of the ASFV-WT-C group, ranging from 2.95 to 6.22 Log_10_copies/mL, while no viral DNA was detected in the tissue samples of two live pigs in the ASFV-ΔE120R immunization group (Figure 8(F)). To further evaluate the activation of cellular immune responses in surviving pigs, immunofluorescence double staining for CD8 and IFN-γ was performed on lung and spleen tissues from the Mock, ASFV-WT, and ASFV-ΔE120R groups. Quantification of CD8^+^IFN-γ^+^ colocalized areas revealed that the ASFV-ΔE120R group surviving pigs exhibited a significantly greater extent of colocalization in both the lung and spleen compared to the ASFV-WT and Mock groups, indicating that ASFV-ΔE120R effectively induces functional activation of CD8^+^ T cells in these tissues (Figure S3E-H). The mRNA expression levels of *IFN-α*, *TNF-α*, *ISG15*, *ISG54*, *ISG56*, and *MX1* in the liver and bone marrow of ASFV-WT-C pigs and the surviving ASFV-ΔE120R-immunized pigs were quantified by RT-qPCR. Results demonstrated significantly higher expression of these mRNA level in both liver and bone marrow of ASFV-ΔE120R-immunized survivors compared to ASFV-WT-C group pigs (Figure S3I-J). Taken together, these findings indicate that ASFV-ΔE120R immunization confers partial protection against homologous viral challenge in swine.

### ASFV-ΔE120R protects piglets from pathological damage caused by parental strain challenge

**Figure 9.**
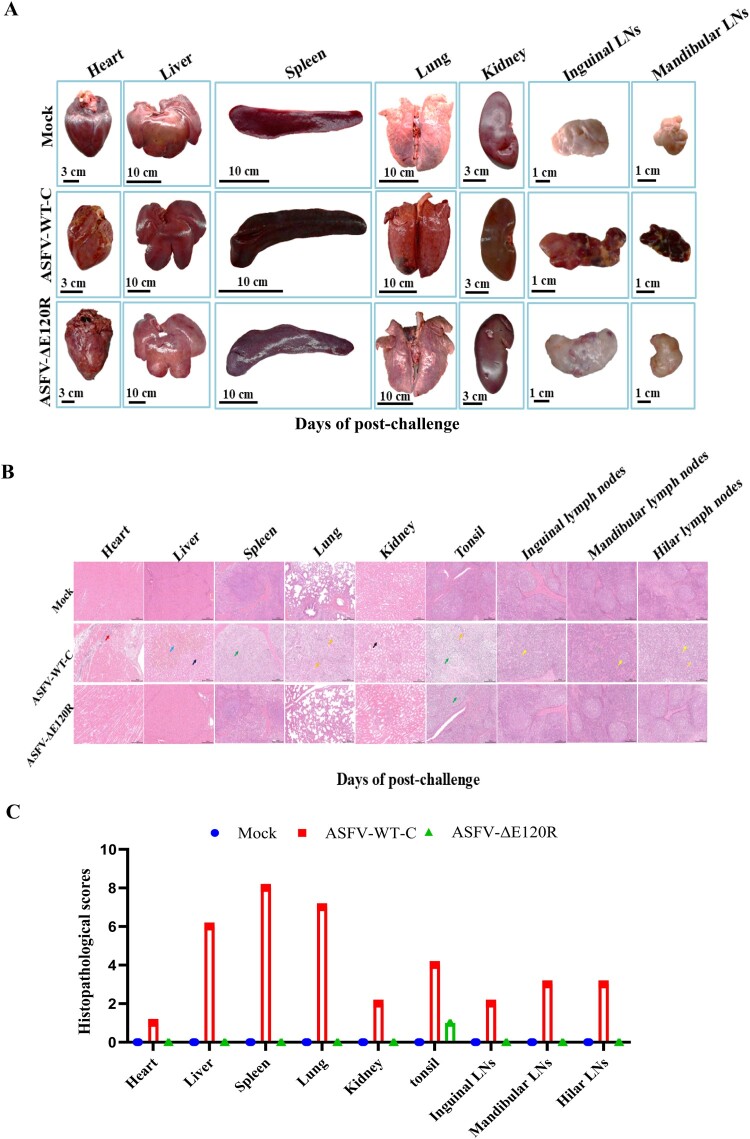
Histopathological analysis of tissue damage in the surviving ASFV-ΔE120R-immunized pigs and ASFV-WT-C group pigs. (A) Gross pathological features of the heart, liver, spleen, lung, kidney, mandibular lymph nodes, and inguinal lymph nodes in the surviving ASFV-ΔE120R-immunized pigs and ASFV-WT-C group pigs. (B) Histopathological changes in the heart, liver, spleen, kidney, tonsil, inguinal lymph nodes, mandibular lymph nodes, and hilar lymph nodes of surviving immunized pigs and ASFV-WT-C group pigs. ASFV-WT-C: heart myofiber necrosis and lysis with inflammatory infiltration (↑); liver hepatocyte necrosis with extensive hemorrhage (↑) and sinusoidal congestion (↑); spleen white pulp atrophy, indistinct demarcation between white and red pulp, and lymphocyte necrosis (↑); lung alveolar and bronchial lumina filled with inflammatory cells and necrotic debris, loss of normal alveolar architecture (↑); kidney proteinaceous casts (↑); tonsil lymphocyte depletion (↑) and inflammatory cell infiltration in crypts (↑); inguinal lymph nodes loss of lymphoid follicles and lymphocyte depletion (↑); mandibular lymph nodes congestion (↑) and reduced lymphoid follicles (↑). ASFV-ΔE120R: tonsil focal lymphocyte necrosis and mild lymphocyte depletion within lymphoid follicles (↑). (C) Histopathological scores of the heart, liver, spleen, lung, kidney, tonsil, inguinal lymph nodes, mandibular lymph nodes, and hilar lymph nodes in the surviving ASFV-ΔE120R-immunized pigs and ASFV-WT-C group pigs.

ASFV infection induces pathological changes characterized by multi-organ enlargement and haemorrhagic necrosis, particularly splenomegaly and lymph node haemorrhage [10]. Post-mortem analysis of experimental animals compared tissue lesions between the surviving ASFV-ΔE120R-immunized pigs and ASFV-WT-C group pigs. ASFV SY18 infection caused severe organ damage, including extensive haemorrhages in the heart and liver; enlarged, friable, and darkened spleen; and haemorrhagic necrosis in the lung, kidney, and lymph nodes. In contrast, no gross pathological lesions were observed in the surviving ASFV-ΔE120R-immunized pigs (Figure 9(A)). Haematoxylin–eosin (HE) staining further revealed fibroplasia in cardiac tissue, necrotic haemorrhage in hepatic tissue, white pulp atrophy in splenic tissue, inflammatory cell infiltration in pulmonary tissue, proteinaceous casts in renal tissue, and lymphocyte depletion in tonsil and lymph node tissues of ASFV-WT-C pigs. Notably, the surviving immunized pigs exhibited only focal lymphocyte necrosis and mild lymphocyte depletion in tonsillar lymphoid follicles, with no other histopathological abnormalities (Figure 9(B,C)). These findings demonstrate that the surviving ASFV-ΔE120R-immunized pigs exhibited significantly reduced histopathological damage compared to ASFV-WT-C pigs following challenge with the parental ASFV SY18 strain.

## Discussion

The ASFV genome encodes over 150 proteins that orchestrate multifaceted roles in viral propagation and immune evasion, yet the mechanistic underpinnings of many remain poorly defined. Targeted deletion of immune evasion-associated genes has emerged as a cornerstone strategy for engineering live-attenuated ASFV vaccines. In this study, we demonstrate that the E120R gene-deleted strain (ASFV-ΔE120R) not only attenuates pathogenicity by subverting canonical virion release dynamics but also distinguishes itself as a promising vaccine candidate through its capacity to provoke robust host immune activation.

The E120R gene-deleted strain was successfully constructed and purified through homologous recombination coupled with limited dilution, confirming that the E120R gene is non-essential for viral replication – a conclusion consistent with findings by GERMA´N ANDRE´S et al. [15]. Biological characterization revealed significantly reduced replication efficiency of ASFV-ΔE120R compared to the parental strain ASFV SY18. TEM demonstrated that E120R deletion resulted in the formation of aberrant tubular structures alongside normal icosahedral virions in PAMs, a phenotype analogous to that observed in H240R-deleted ASFV [21]. Notably, these tubular structures may enhance immunogenicity by exposing internal viral antigenic epitopes. Furthermore, our study suggests that the deletion of E120R impairs viral release, which may be attributed to a disruption in the virus budding process [15]. While ASFV-WT acquires an outer envelope via host cell membrane budding [22]. ASFV-ΔE120R may fail to obtain this intact envelope due to impaired budding. As a result, ASFV-ΔE120R particles released via cell lysis may differ in both structure and antigenicity from those of ASFV-WT. Additionally, deletion of the E120R gene may interfere with proper virion assembly, potentially altering the exposure of key antigenic epitopes. These structural and assembly defects could contribute to the increased sensitivity of ASFV-ΔE120R to neutralization by convalescent pig sera.

To ensure survival and progeny production, ASFV encodes diverse proteins to subvert host immune defenses. For instance, the DP96R protein inhibits TBK1 phosphorylation mediated by the cGAS/STING signalling pathway [23], while A276R, A528R, I329L, and MGF360-12L suppress IFN-β production to dampen antiviral immunity [24–26]. Previous studies have also implicated E120R in attenuating host antiviral responses through cGAS/STING pathway suppression [19,20]. Building on this foundation, RNA-seq analysis of ASFV-ΔE120R-infected PAMs revealed that the E120R-deleted strain upregulated the expression of antiviral factors, including *CCL5*, *ISG15*, *MX1*, and *JAK2*, compared to ASFV-WT. Concurrently, ASFV-ΔE120R activated inflammatory and innate immune signalling pathways. RT-qPCR further validated the elevated expression of IFN-associated antiviral effectors such as *IFN-α*, *ISG15*, *ISG54*, *ISG56*, and *MX1* in ASFV-ΔE120R-infected PAMs. These findings demonstrate that E120R deletion relieves its suppressive effects on host IFN signalling, thereby potentiating the activation of antiviral defenses. Given the critical role of IFN signalling as an innate immune barrier against ASFV infection [27], the enhanced expression of IFN-related factors not only corroborates the immune evasion function of pE120R but also suggests that ASFV-ΔE120R may exhibit superior immunogenicity through amplified activation of innate immune responses. Although RNA-seq and RT-qPCR analyses in our study suggest that deletion of E120R enhances host antiviral responses – including upregulation of interferon-stimulated genes and chemokines – our investigation did not include direct molecular assays such as co-immunoprecipitation, reporter assays, or domain mapping to elucidate the precise mechanisms by which pE120R modulates innate immunity. Previous studies by Liu et al demonstrated that pE120R inhibits IRF3 phosphorylation via direct interaction, while Cui et al reported that pE120R suppresses the cGAS-STING and NF-κB signalling pathways, thereby inhibiting IFN-β production [19,20]. These findings provide valuable insights into the mechanistic roles of pE120R; however, our study focused on evaluating the immunological and virological consequences of E120R deletion in a virulent genotype II ASFV background. We acknowledge that the downstream effects of pE120R-host protein interactions, such as IRF3 nuclear translocation and transcriptional activation, were not addressed here. Future work incorporating direct protein-interaction assays and functional domain analysis will be essential to build upon our current findings and further clarify how pE120R contributes to ASFV immune evasion.

Gene deletion represents a validated approach for developing live-attenuated ASFV vaccines. Single or multi-gene deletions have been shown to confer protection during ASFV infection [28,29]. For instance, deletions in I177L, I226R, I73R, or 9GL attenuate the virulence of highly pathogenic ASFV strains and protect against parental virus challenge [30-33]. Similarly, removal of MGF family genes (e.g. MGF-360-10L, MGF-110-9L, MGF-505-7R) from virulent ASFV isolates reduces pathogenicity in domestic pigs and induces protective immunity [34–36]. In this study, pigs immunized with ASFV-ΔE120R exhibited no detectable viral DNA in heart, liver, spleen, lung, kidney, tonsil, lymph nodes (mandibular, inguinal, mesenteric), bone marrow, or muscle at 4, 7, 10, or 14 days post-immunization During a 28-day observation period, an independent cohort of pigs immunized with ASFV-ΔE120R survived without clinical abnormalities, maintaining normal rectal temperatures. Critically, no viral genome copies were detected in blood, oropharyngeal swabs, nasal swabs, or anal swabs from immunized or contact-exposed pigs. The absence of viral shedding may stem from structural alterations in ASFV-ΔE120R virions, which could enhance neutralization by host antibodies, thereby limiting replication, or result from viral excretion levels below qPCR detection thresholds. This contrasts with previous reports of viral shedding in pigs immunized with other deletion strains [2,6,32,37], including recent findings that the commercially developed ASFV-G-ΔI177L strain caused moderate ASF-related clinical signs in a pregnant sow and the live-born piglets were infected with ASFV [4]. Thus, the shedding of attenuated ASFV strains and subsequent environmental contamination has long been a critical issue requiring attention and resolution. The unique characteristics of ASFV-ΔE120R effectively address this major limitation, thereby enhancing its potential as a vaccine candidate.

Humoral and cellular immunity analyses revealed anti-p54 antibody levels (S/P ratio: 0.05–0.62) in ASFV-ΔE120R-immunized pigs at 21 dpi, lower than those reported for other deletion strains [38–41]. Booster immunization elevated antibody titres to 0.31–1.14 (S/P ratio) pre-challenge. All immunized pigs developed cellular immunity within 28 days – a dominant mediator of protection in ASFV vaccination [42]. Post-challenge with the parental ASFV SY18 strain, partial protection (2/5 survival) was observed. The two surviving pigs displayed no ASF-specific clinical signs, except for transient hyperthermia (40.5°C) observed in one pig, and exhibited elevated anti-p54 antibody levels and increased numbers of IFN-γ-secreting PBMCs. In contrast, three immunized pigs succumbed to infection after developing severe ASF symptoms (persistent fever >40.5°C, tremors, paralysis, anorexia), and no significant increase in anti-p54 antibody levels and numbers of IFN-γ-secreting PBMC cells. Post-mortem analysis of survivors confirmed the absence of viral DNA and no significant histopathological lesions in all sampled tissues. Notably, enhanced functional activation of CD8^+^ T cells were observed in the lungs and spleens, accompanied by elevated IFN-γ secretion, suggesting a stronger cellular immune response. In addition, increased mRNA expression levels of *IFN-α*, *TNF-α*, *ISG15*, *ISG54*, *ISG56*, and *MX1* were detected in the liver and bone marrow of the two surviving pigs. Together, the ELISpot and tissue immunofluorescence results suggest that stronger cellular immune response may contribute to viral clearance and clinical protection. Importantly, the failure of p54-specific antibodies to protect three immunized pigs suggests that anti-p54 responses are not central to immune protection [43]. These outcomes highlight the need for optimized immunization protocols, such as adjuvant co-administration, to enhance ASFV-ΔE120R’s protective efficacy [44].

In conclusion, our study is the first to use the ASFV-E120R deletion mutant as a research model, confirming that viral particle release was impaired and that the resulting virions displayed abnormal morphology, which induced a stronger cellular immune response in the host. Furthermore, the non-Virus shedding characteristics and partial protective efficacy of ASFV-ΔE120R provide new ideas for the development of safe and effective attenuated ASFV vaccines.

## Materials and methods

### Animal ethics statement and experimental procedures

All animal experiments were approved by the Animal Welfare and Ethics Committee of the Changchun Veterinary Research Institute (Review ID: IACUC of CAS-12-2021-011). ASFV propagation and animal studies were conducted in an Animal Biosafety Level 3 (ABSL-3) laboratory. Safety Assessment of ASFV-ΔE120R: Twelve pigs were intramuscularly (i.m.) inoculated with ASFV-ΔE120R (5 × 10^6^ TCID_50_). Three pigs per group were euthanized at 4, 7, 10, and 14 days post-immunization for tissue collection (heart, liver, spleen, lung, kidney, tonsil, inguinal lymph nodes, mandibular lymph nodes, mesenteric lymph nodes, muscle, and bone marrow). Viral DNA in tissues was quantified by qPCR. Tissue homogenates from heart, liver, spleen, lung, kidney, tonsil, lymph nodes (inguinal, mandibular, mesenteric), muscle, and bone marrow were filtered and passaged twice (5 days per passage) in BMDMs. Number of fluorescence-positive cells were documented, and viral DNA in cell cultures was analysed.

Protective Efficacy Evaluation: Pigs were divided into four groups: ASFV-ΔE120R group (n = 5, 5 × 10^6^ TCID_50_, i.m.); Sentinel group (n = 2, contact-exposed pigs); ASFV-WT group (n = 3, 10^2^ TCID_50_, i.m.); Mock group (n = 3, 1 mL RPMI 1640, i.m.). At 21 dpi, the ASFV-ΔE120R group received a booster immunization (5 × 10^6^ TCID_50_, i.m.). Rectal temperatures and clinical signs were monitored daily from day 0, with clinical scoring performed as described in Table S4. Blood samples, oropharyngeal swabs, nasal swabs, and anal swabs were collected from all pigs at 0, 3, 5, 7, 9, 11, 13, 15, 17, 19, 21, 23, 25, and 28 dpi. At 28 dpi, ASFV-ΔE120R-immunized pigs and ASFV-WT-C (n = 2) were challenged with 10 TCID_50_ of the parental ASFV SY18 strain (i.m.). Post-challenge, rectal temperatures and clinical signs were monitored daily. Blood and swab samples were collected at 0, 3, 5, 7, 9, 14, 21, 28, and 35 dpc. At the end of the 35-day observation period, samples of the heart, liver, spleen, lungs, kidneys, tonsils, inguinal lymph nodes, submandibular lymph nodes, hilar lymph nodes, muscle, and bone marrow were collected from the pigs for qPCR detection of viral load. In addition, heart, liver, spleen, lungs, kidneys, tonsils, inguinal lymph nodes, submandibular lymph nodes, and hilar lymph nodes from the surviving pigs were collected for HE staining and histopathological analysis. RNA was extracted from the spleen and bone marrow of the pigs to assess the mRNA levels of antiviral factors including *IFN-α*, *TNF-α*, *ISG15*, *ISG54*, *ISG56*, and *MX1*.

### Cell culture and viruses

PAMs were isolated via bronchoalveolar lavage, and BMDMs were differentiated from porcine bone marrow. Cells were cultured in RPMI 1640 medium (Gibco, USA) supplemented with 10% fetal bovine serum (FBS) and maintained at 37°C under 5% CO_2_ incubator. The DNA of African swine fever virus, classical swine fever virus (CSFV), porcine reproductive and Respiratory syndrome virus (PRRSV), pseudorabies virus (PRV), porcine parvovirus and porcine circovirus 1/2 (PCV1/2) was tested in accordance with national standards to ensure that the pigs used in the experiments were not infected with any porcine related viruses.

The ASFV SY18 strain (GenBank accession no. MH766894) was isolated from clinical samples during an ASF outbreak. The recombinant ASFV-ΔI267L strain was constructed by replacing the *I267L* gene with the enhanced green fluorescent protein (eGFP) gene in the parental ASFV SY18 backbone.

### Construction of the E120R gene-deleted ASFV strain

The recombinant ASFV-ΔE120R was generated via homologous recombination using the parental ASFV SY18 genome and a recombinant transfer vector, as previously described [31]. Briefly, the 1.2 kb left homologous arm (nucleotides 166,677–167,876) and 1.2 kb right homologous arm (nucleotides 168,135–169,334) of the *E120R* gene were fused by PCR and flanked to the p72 promoter-driven mCherry reporter cassette in the plasmid backbone, as illustrated in Figure 1A. The deleted E120R gene spans nucleotides 167,876–168,135 in the full-length genome of ASFV SY18. The recombinant plasmid was transfected into BMDMs, followed by infection with ASFV SY18. Recombinant viruses were identified by fluorescence microscopy 24 h post-infection. Purified ASFV-ΔE120R was obtained through limited dilution, amplification culture, and purity verification. To further eliminate residual host cell debris, the LDS-L2 ultrafiltration system (AITESEN, Suzhou, China) was used during the purification process.

### Whole-Genome sequencing and analysis of ASFV

To validate the genomic integrity of the recombinant virus, the full-length genome sequence was determined by next-generation sequencing (NGS). Total DNA was extracted from cell culture-derived virions, and 1 μg of DNA was subjected to sequencing using the Illumina NovaSeq 6000 platform with paired-end 150 bp (PE150) read configuration (Novogene, Tianjin, China).

### One-step virus growth kinetics and TCID₅₀ Titration

PAMs were infected with either ASFV-WT (ASFV SY18) or ASFV-ΔE120R at a multiplicity of infection of 0.01. Total cell cultures (i.e. combined supernatant and cell pellet) were collected from infected cells at 0, 12, 24, 48, 72, 96, and 120 hpi. Viral titres at each time point were determined by the TCID₅₀ assay to generate virus growth curves. PAMs were cultured in 96-well plates and infected with 10-fold serial dilutions of ASFV-WT or ASFV-ΔE120R, with eight replicates for each dilution. For cells infected with ASFV-WT or ASFV-ΔE120R, infection was detected by staining with a fluorescein isothiocyanate (FITC)-conjugated anti-p72 monoclonal antibody (prepared in-house) and observing fluorescence-positive cells under a fluorescence microscope. Viral titres were calculated using the Reed-Muench method.

### Western blot

Total cellular proteins were extracted using RIPA lysis buffer supplemented with EDTA and phosphatase inhibitors. Protein concentrations were determined using a BCA Protein Assay Kit (KeyGEN, China). Equal amounts of protein were separated by SDS-PAGE and transferred onto PVDF membranes. The membranes were blocked with 5% non-fat milk at room temperature for 1 h and then incubated overnight at 4°C with the following primary antibodies: anti-β-actin (1:3000 dilution; Beyotime, China), anti-p30 (1:1000 dilution; prepared in-house), and anti-pE120R (1:1000 dilution; prepared in-house). After six washes with TBST (5 min each), membranes were incubated with a HRP-conjugated goat anti-rabbit IgG secondary antibody (1:3000 dilution; Beyotime, China) at room temperature for 1 h, followed by three washes with TBST (10 min each). Protein expression levels were detected using the Tanon 5200 imaging system (Tanon5200, China), and band intensities were quantified using ImageJ software.

### Transmission electron microscopy (TEM)

PAMs were infected with either ASFV-WT or ASFV-ΔE120R at a MOI of 1 and harvested at 12, 24, and 48 hpi. Cells were collected by centrifugation at 1000 rpm for 10 min and fixed with 2.5% glutaraldehyde at 4°C for 12 h. The fixed cell pellet, approximately the size of a small green bean, was processed for sectioning and placed onto copper grids. Samples were subsequently stained with 2% osmium tetroxide and lead citrate. Ultrathin sections were observed and imaged using a transmission electron microscope (Hitachi, TEM-HT7700).

### Virus release assay

PAMs were infected with either ASFV-WT or ASFV-ΔE120R at a MOI of 1. After 2 h of viral adsorption, the inoculum was removed, and the cells were washed twice with PBS. The infected cells were then cultured, and samples were collected at 2, 6, 12, 24, 36, and 48 hpi. In cells, supernatants, and in total (pellet and supernatants) were harvested separately. Viral titres were subsequently determined for cells, supernatants, and in total (pellet and supernatants).

### Serum neutralization assay

Equal volumes of ASFV-WT, ASFV-ΔI267L, or ASFV-ΔE120R virus (100 TCID₅₀) were mixed with serum samples previously diluted 1:10 in RPMI 1640 medium. For positive controls, equal volumes of ASFV-WT, ASFV-ΔI267L, or ASFV-ΔE120R virus (100 TCID₅₀) were mixed with negative serum samples. For negative controls, RPMI 1640 medium was mixed with negative serum samples. After incubation at 37°C for 2 h, the mixtures were added to PAMs seeded in 96-well plates and cultured at 37°C in a 5% CO₂ incubator for 5 days. On day 5, fluorescence was observed and images were captured. Whole cell culture were collected for viral genome quantification by qPCR and virus titration by TCID₅₀ assay.

### RNA-Seq analysis

PAMs were infected with either ASFV-WT or ASFV-ΔE120R at a MOI of 1, and cells were harvested at 4, 12, and 20 hpi for RNA extraction. The extracted RNA was sent to Novogene for RNA-seq analysis (Tianjin, China).

### Reverse transcription quantitative polymerase chain reaction (RT-qPCR)

Total RNA was extracted from PAMs and porcine tissue samples using TRIzol reagent (Thermo Fisher Scientific, USA). Reverse transcription was performed using the PrimeScript FAST RT Reagent Kit with gDNA Eraser (Takara, Dalian, China). RT-qPCR was conducted with TB Green (Takara, Dalian, China). GAPDH was used as an internal control to normalize and quantify the expression of target genes. Relative gene expression levels were calculated using the 2^^-ΔΔCt^ method. Primer sequences are listed in Table S5.

### Quantitative polymerase chain reaction (qPCR)

ASFV genomic DNA was extracted from cell culture supernatants, tissue homogenates, or anticoagulated blood using the FastPure Viral DNA/RNA Mini Kit (Vazyme, China). qPCR was performed using Premix Ex Taq™ (Takara, Dalian, China) on a RocheCycle480 instrument. The target for ASFV genomic amplification was a conserved p72 gene fragment (primers listed in Table S5). The amplification conditions were as follows: 30 s of preheating at 95°C; 40 cycles of 5 s at 95°C, 30 s of annealing at 58°C, and extension at 72°C. ASFV genome copy numbers in samples were calculated using a standard curve derived from a plasmid containing the ASFV SY18 B646L gene (p72), with the equation y = −3.445x + 41.19.

### Enzyme-linked immunosorbent assay (ELISA)

An in-house indirect ELISA kit was used to detect p54 antibodies. Briefly, serum samples (S) and positive control (P) were added to the ELISA plate and incubated at room temperature for 1 h. HRP-conjugated sheep anti-pig IgG (CWBIO, Haimen, China) was then added and incubated at room temperature for 1 h. TMB substrate (SeraCare, Delaware, USA) was added to initiate the colour reaction, and 2M sulphuric acid (Beijing, China) was used to stop the reaction. The optical density (OD) at 450 nm was measured using the iMarkTM Microplate Reader (BIO-RAD, USA). The S/P calculation method is as follows: the average OD450 nm of the Negative Control (NC) = (NC1 + NC2), the average OD450 nm of the Positive Control (PC) = (PC1 + PC2) / 2, and S/P of the sample = Sample OD value / PC OD mean. An S/*P* value > 0.25 was considered positive for ASFV p54 antibodies, while an S/*P* value < 0.25 was considered negative.

### Enzyme-linked immunospot assay (ELISpot)

PBMCs were isolated from EDTA-treated whole blood of pigs on day 21 and day 28 post-immunization (Solarbio, China). ELISpot assays were performed according to the manufacturer's instructions (IMMUNOSpot, USA). The spot-forming cells (SFC) were counted using the ELISpot reader system (IMMUNOSpot, USA), and the number of spots per well was converted to the number of spot-forming cells per million cells (SFC/10⁶ cells).

### Haematoxylin–Eosin (H&E) staining

Formalin-fixed tissues were embedded in paraffin and sectioned at 5 μm thickness. Sections were deparaffinized in xylene and rehydrated through graded ethanol series. Samples were then stained with haematoxylin and eosin, followed by dehydration through graded ethanol, clearing in xylene, and mounting with neutral resin. Stained sections were observed and imaged under a light microscope. Histopathological scoring was performed according to Table S6.

### Tissue immunofluorescence analysis of CD8^+^IFN-γ+ cells

Paraffin-embedded tissue sections were deparaffinized in xylene and rehydrated through a graded ethanol series to distilled water. Antigen retrieval was performed in citrate buffer using microwave heating for 20 min. Endogenous peroxidase activity was quenched with 3% hydrogen peroxide for 25 min at room temperature in the dark. Sections were blocked with bovine serum albumin (BSA) for 30 min and incubated overnight at 4°C with IFN-γ antibody (proteintech, USA). After PBS washes, sections were incubated with HRP-conjugated secondary antibody (Servicebio, China) for 50 min at room temperature, followed by tyramide signal amplification (TSA; CY3-Tyramide) for 10 min in the dark. For sequential double staining, a second round of antigen retrieval was performed, followed by incubation with the CD8 rabbit antibody (novusbio, USA) overnight at 4°C, and detection with FITC-conjugated goat anti-rabbit IgG secondary antibody (Servicebio, China) for 30 min at 37°C. Nuclei were counterstained with DAPI for 10 min, and sections were mounted with antifade mounting medium. Use OLYMPUS for image capture (VS200, OLYMPUS, JAPAN).

## Statistical analysis

Statistical analyses were performed using GraphPad Prism 9.0 software (Inc., La Jolla, USA). Differences between experimental and control groups were evaluated by one-way analysis of variance (ANOVA). *P* values < 0.05 were considered statistically significant (*P < 0.05; **P < 0.01; ***P < 0.001; ns, not significant).

## Author contributions

L.B.M, M.F.M, and R.L.H have conceived as well as designed the experiments. L.B.M, Q.Y.J, N.L, J.F.W, Q.L, X.H.Y, Y.Y.Z, and M.F.M have performed the experiments. L.B.M analysed the data. L.B.M contributed reagents/materials/analysis tools. L.B.M and M.F.M wrote the manuscript.

## Supplementary Material

Supplemental Material

Supplementary material_for review.pdf

Figure S2.tif

Figure S1.tif
