# Supplementary figures and images for: Deletion of the African swine fever virus E120R gene completely attenuates its virulence by enhancing host innate immunity and impairing virus release
Source: Emerg Microbes Infect. 2025 Sep 3;14(1):2555722. doi: 10.1080/22221751.2025.2555722 (PMC12451965; doi:10.1080/22221751.2025.2555722)

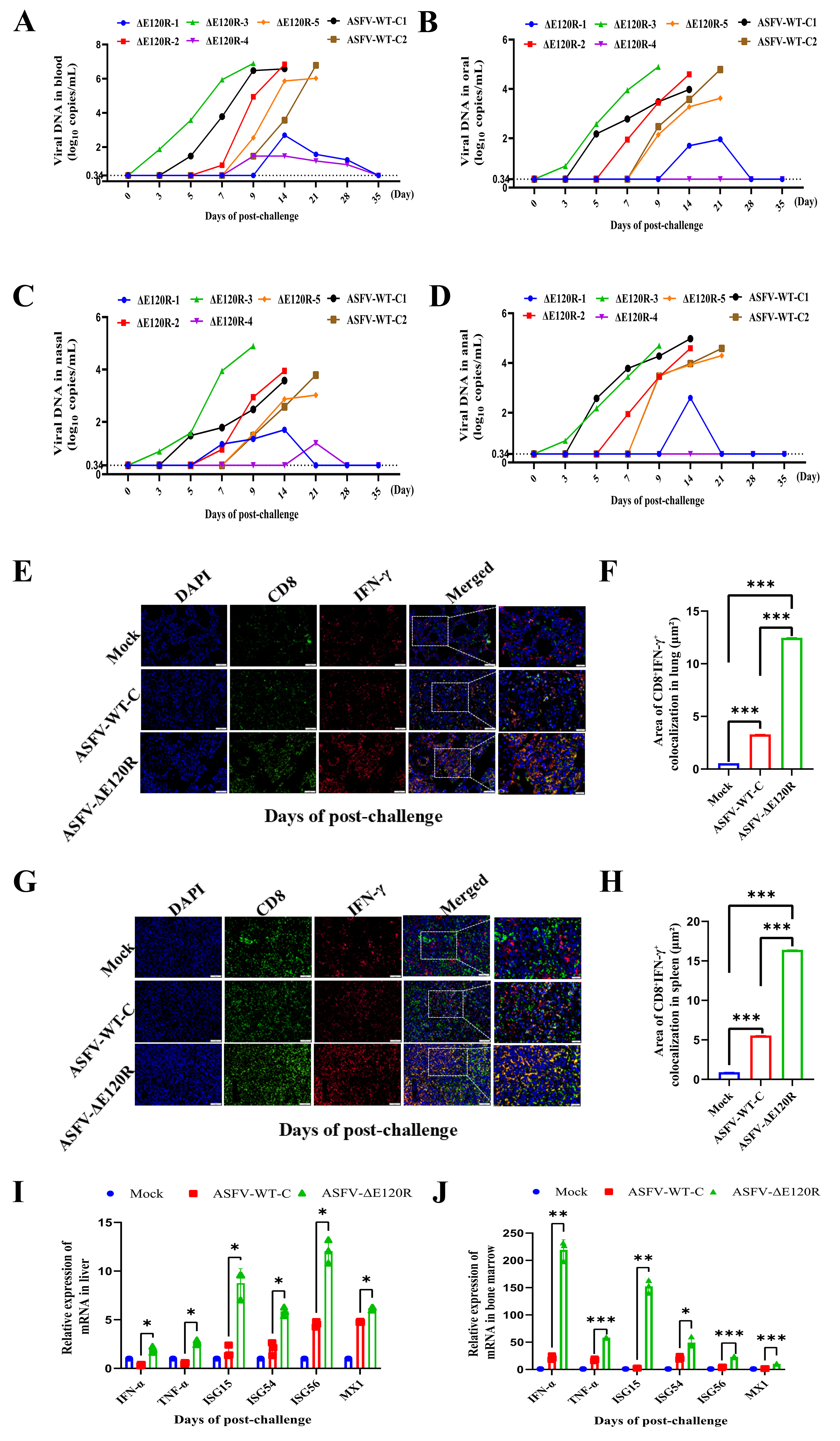

Supplement: Supplemental Material [file TEMI_A_2555722_SM6323.tif]

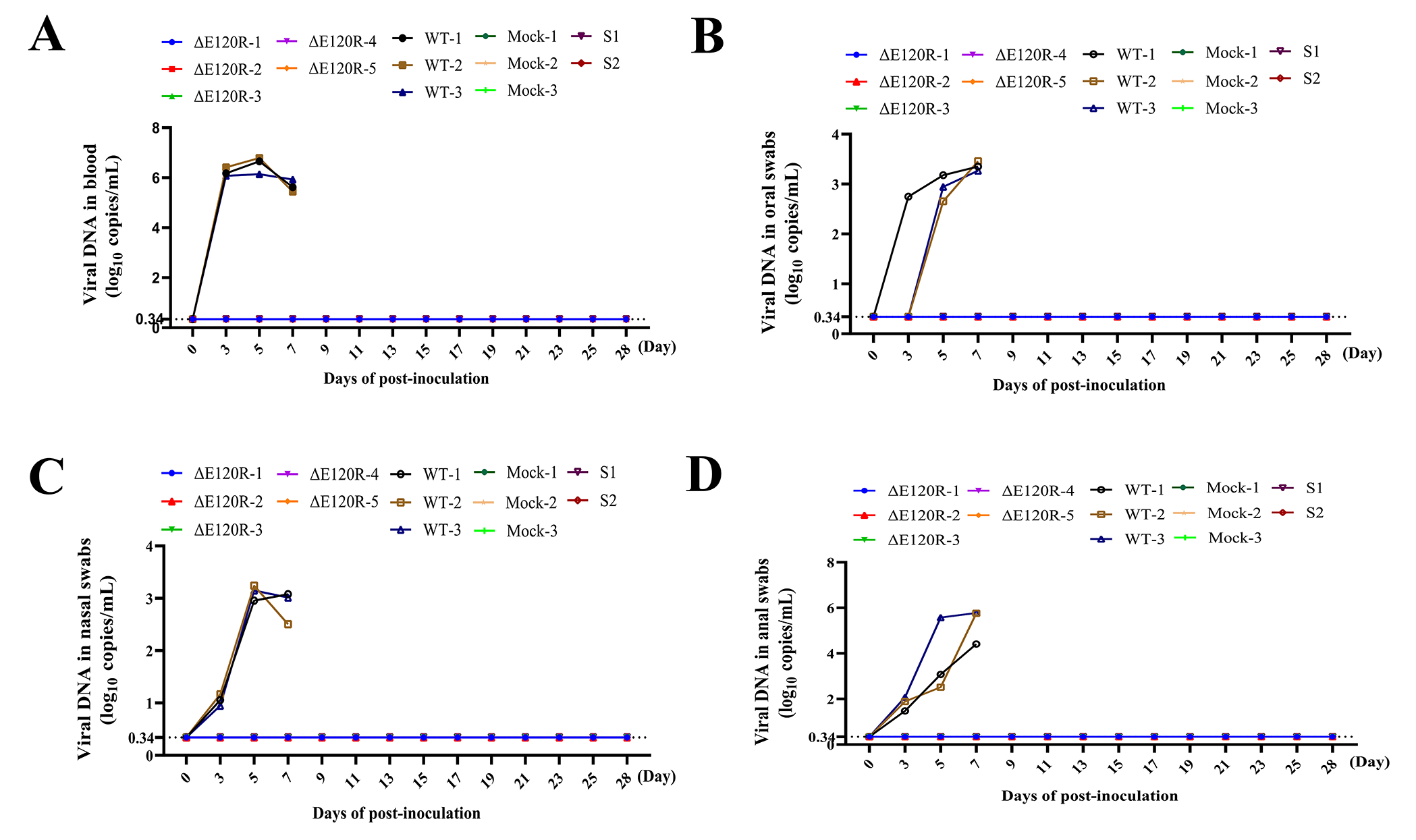

Supplement: Figure S2.tif [file TEMI_A_2555722_SM3198.tif]

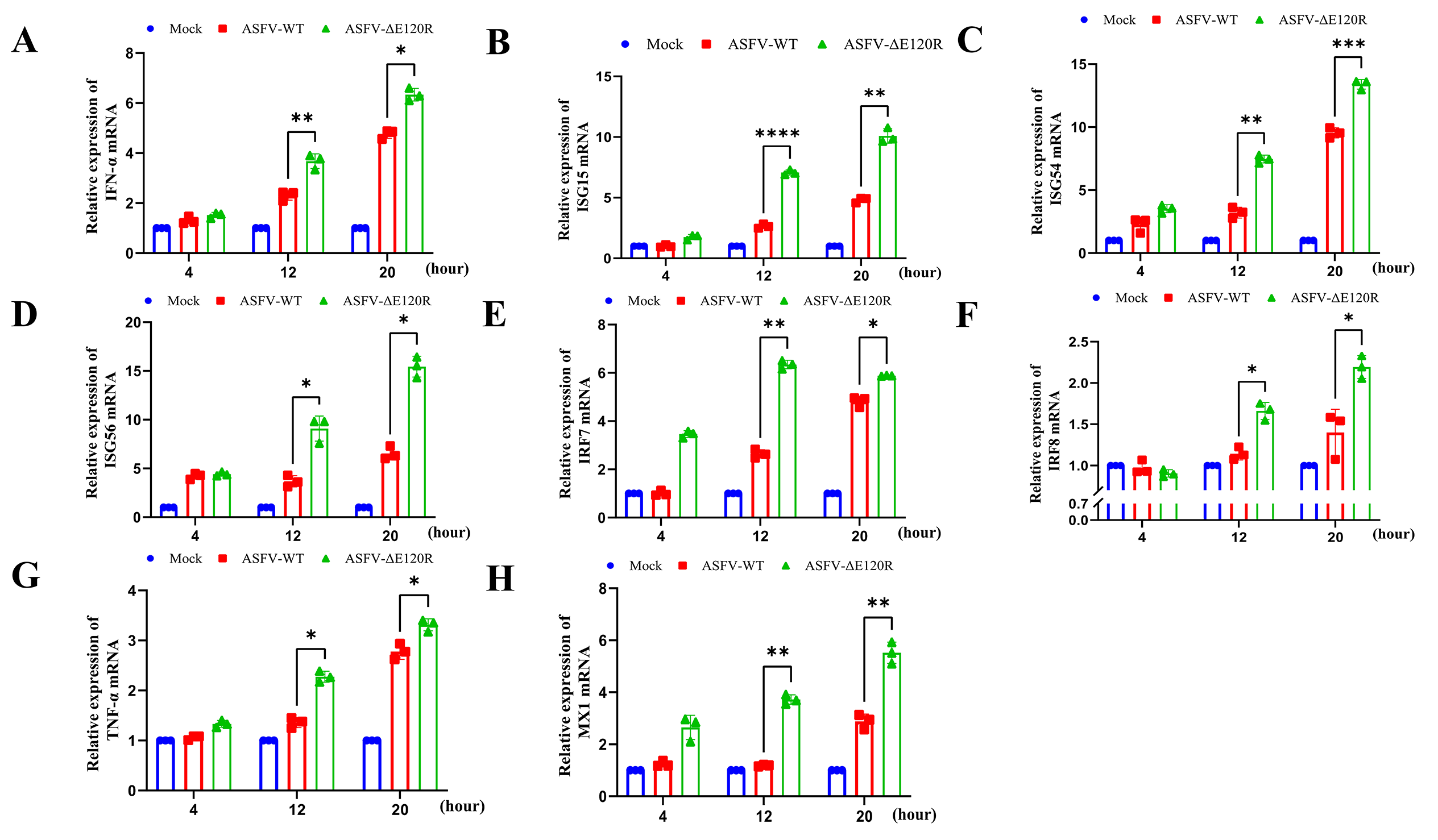

Supplement: Figure S1.tif [file TEMI_A_2555722_SM3197.tif]
