# Supplementary material for: Deletion of the African swine fever virus E120R gene completely attenuates its virulence by enhancing host innate immunity and impairing virus release
Source: Emerg Microbes Infect. 2025 Sep 3;14(1):2555722. doi: 10.1080/22221751.2025.2555722 (PMC12451965; doi:10.1080/22221751.2025.2555722)

ASFV viron budding (ASFV-WT 24 h)

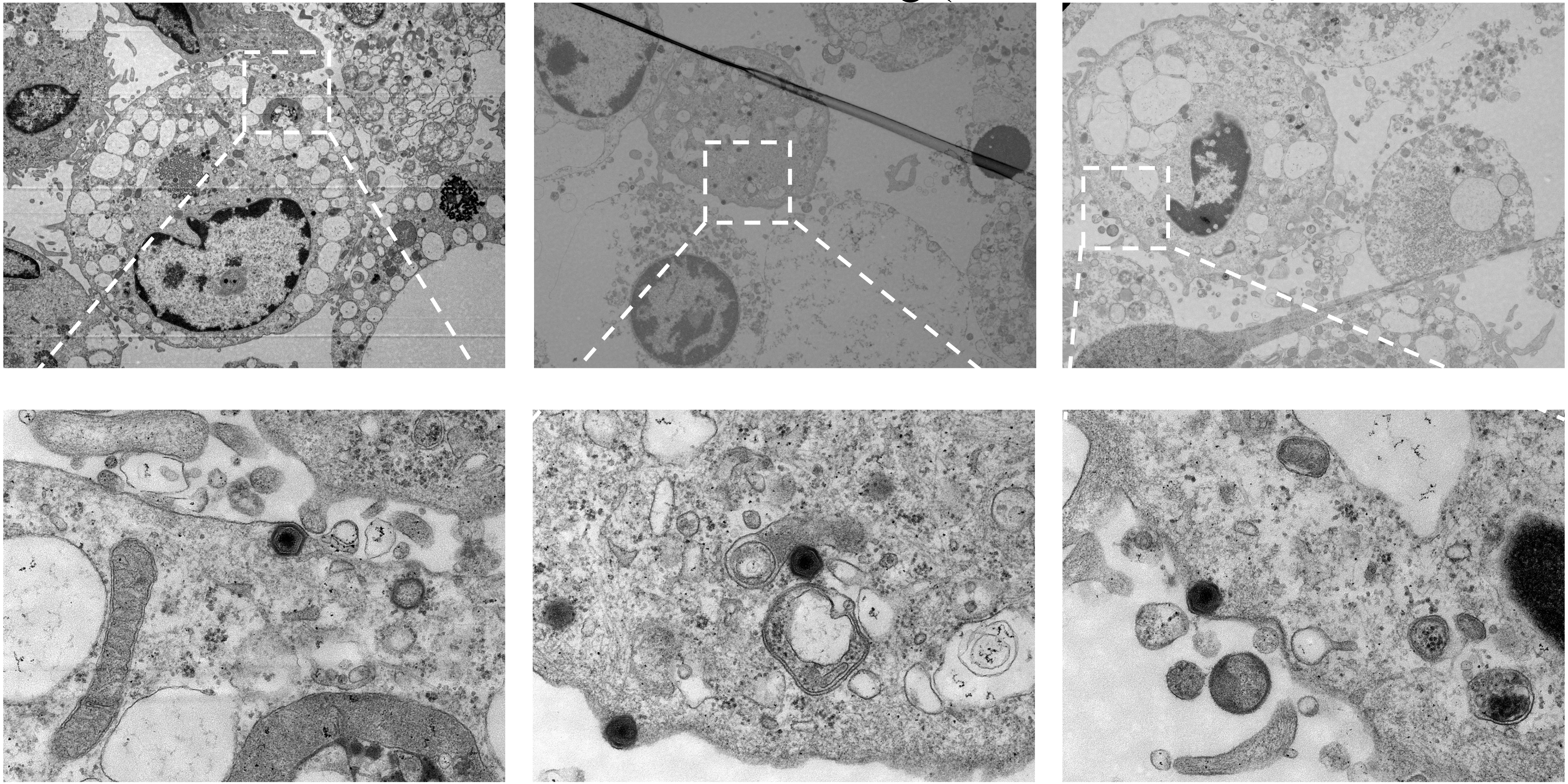

ASFV viron budding (ASFV-WT 24 h)

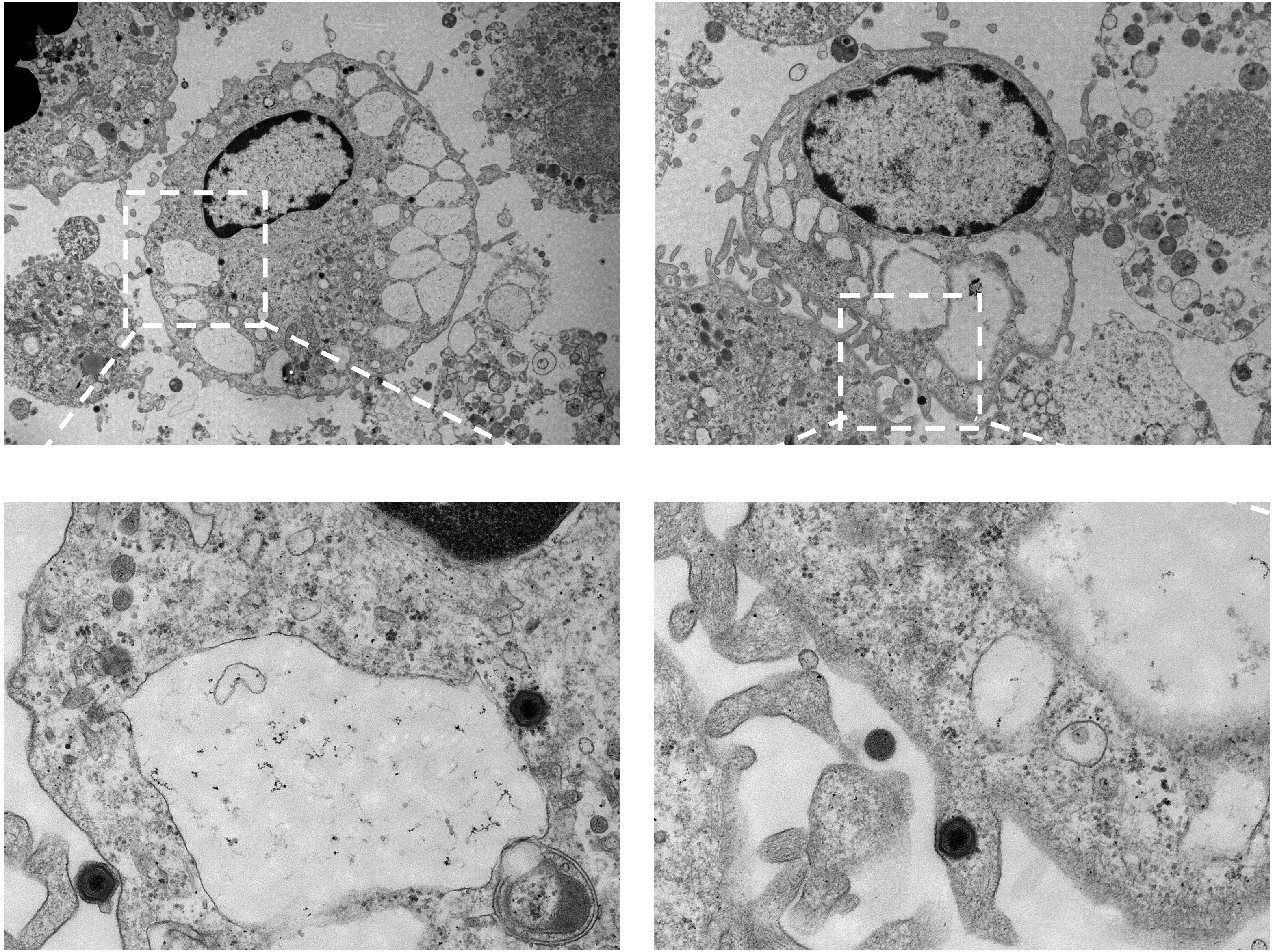

ASFV WT 12 h

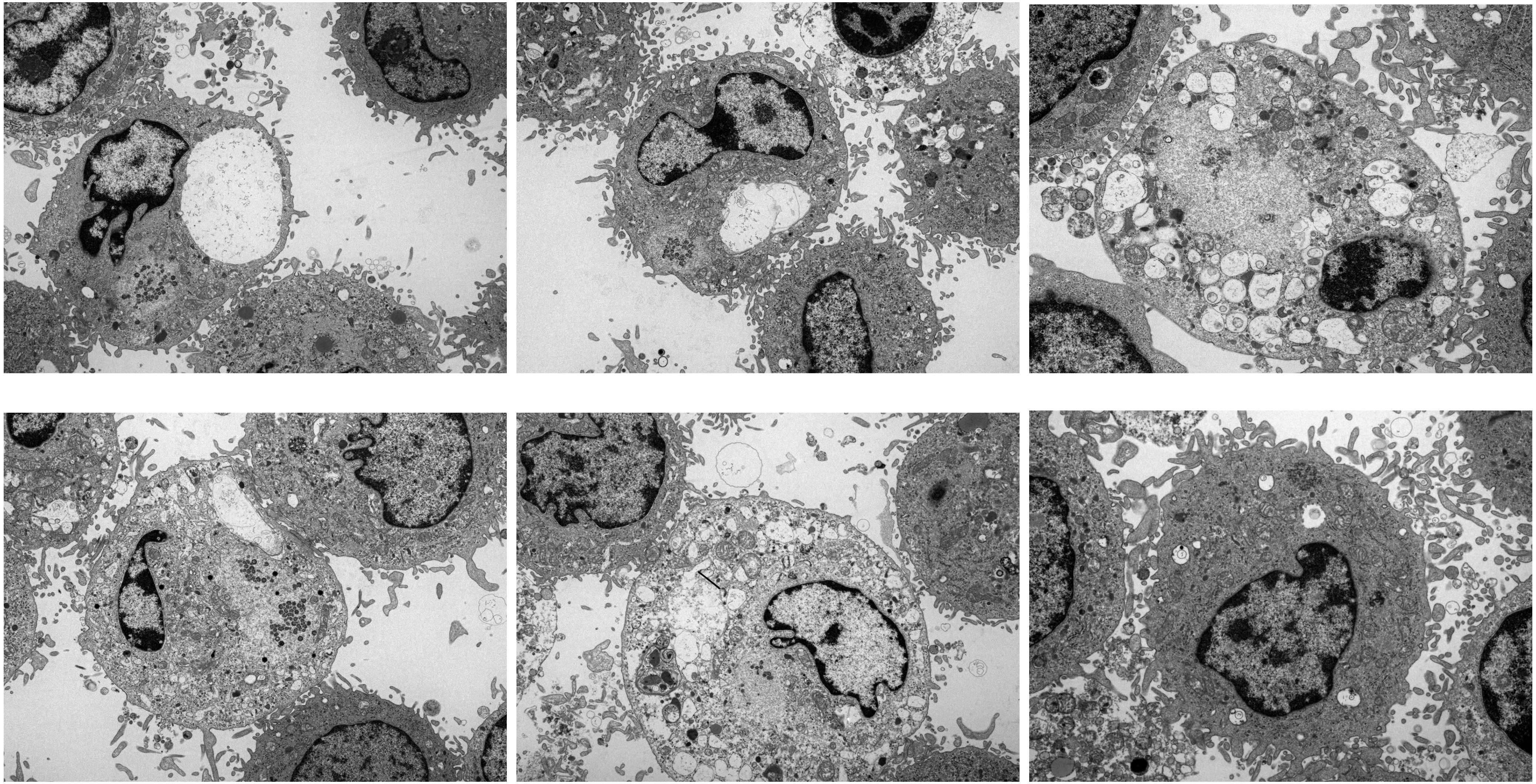

ASFV WT 12 h

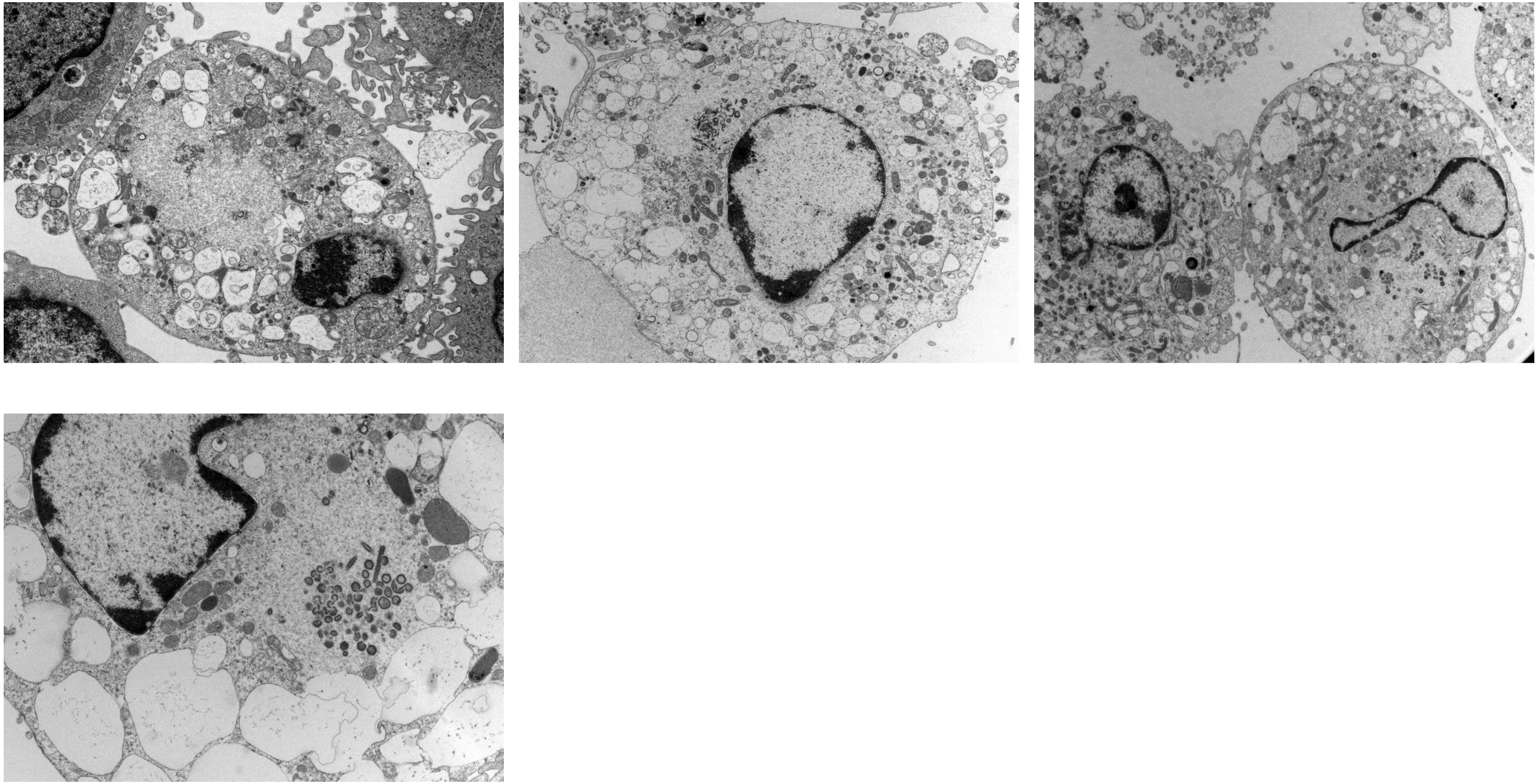

ASFV WT 24 h

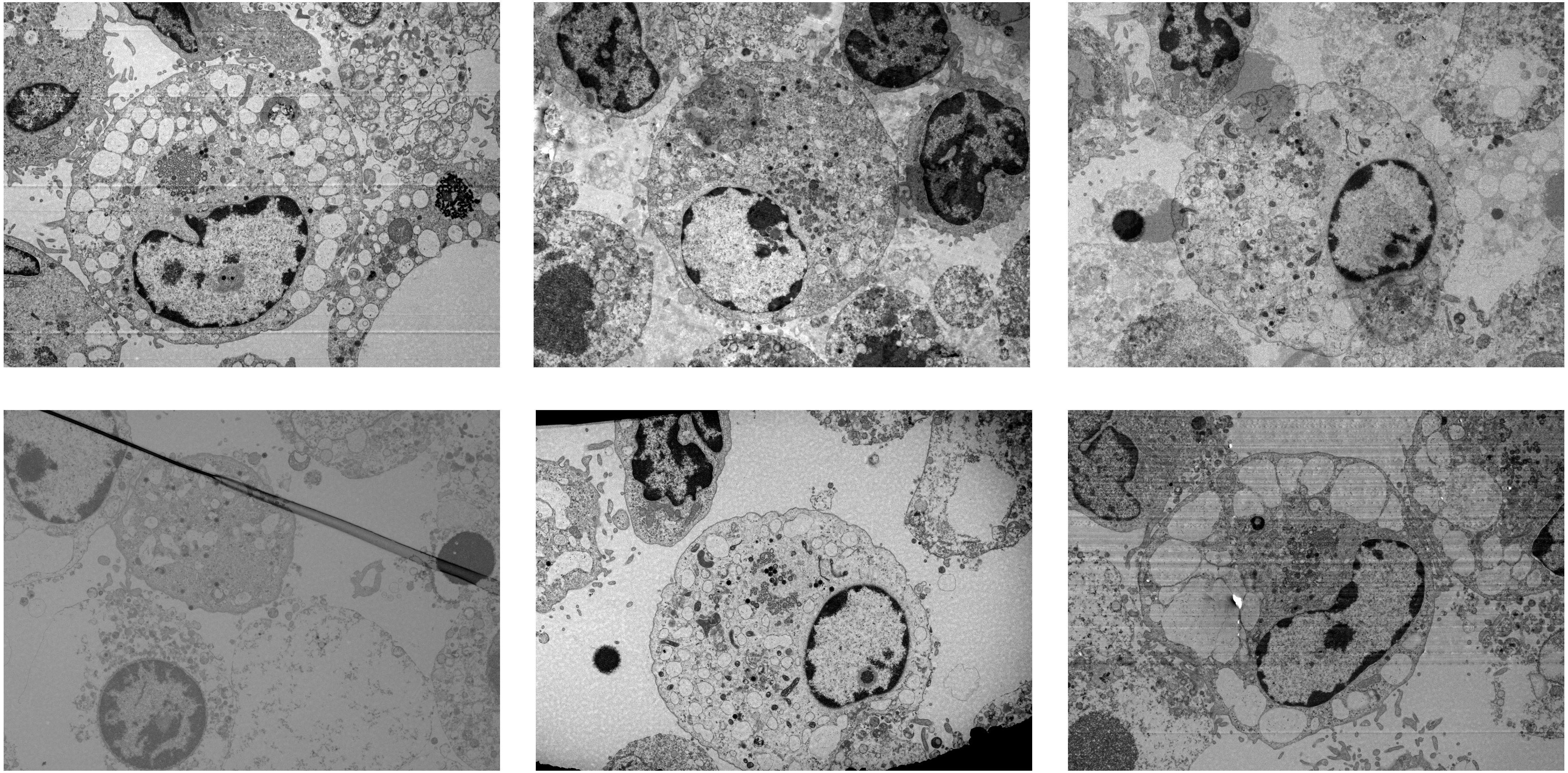

ASFV WT 24 h

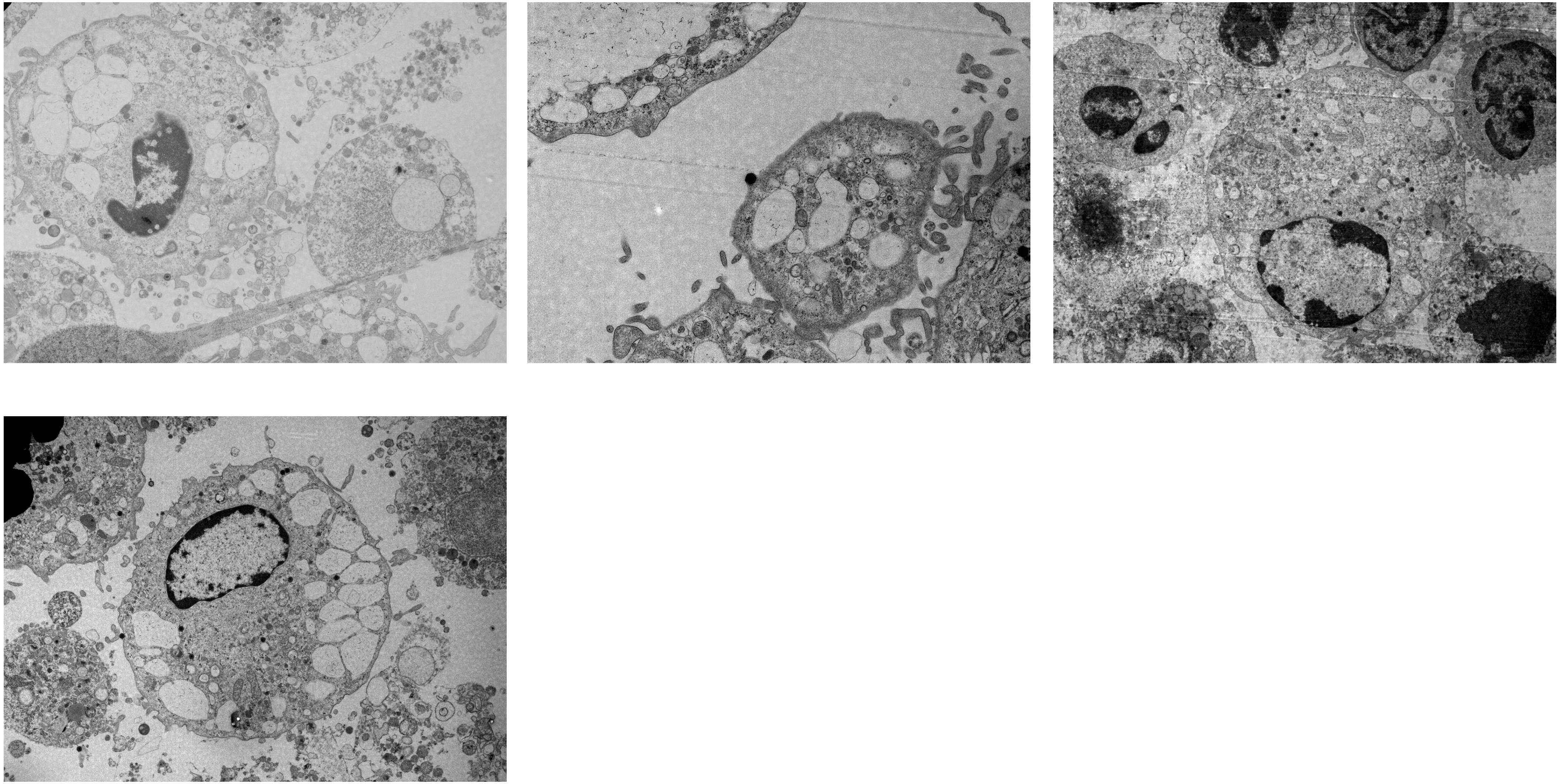

ASFV WT 48 h

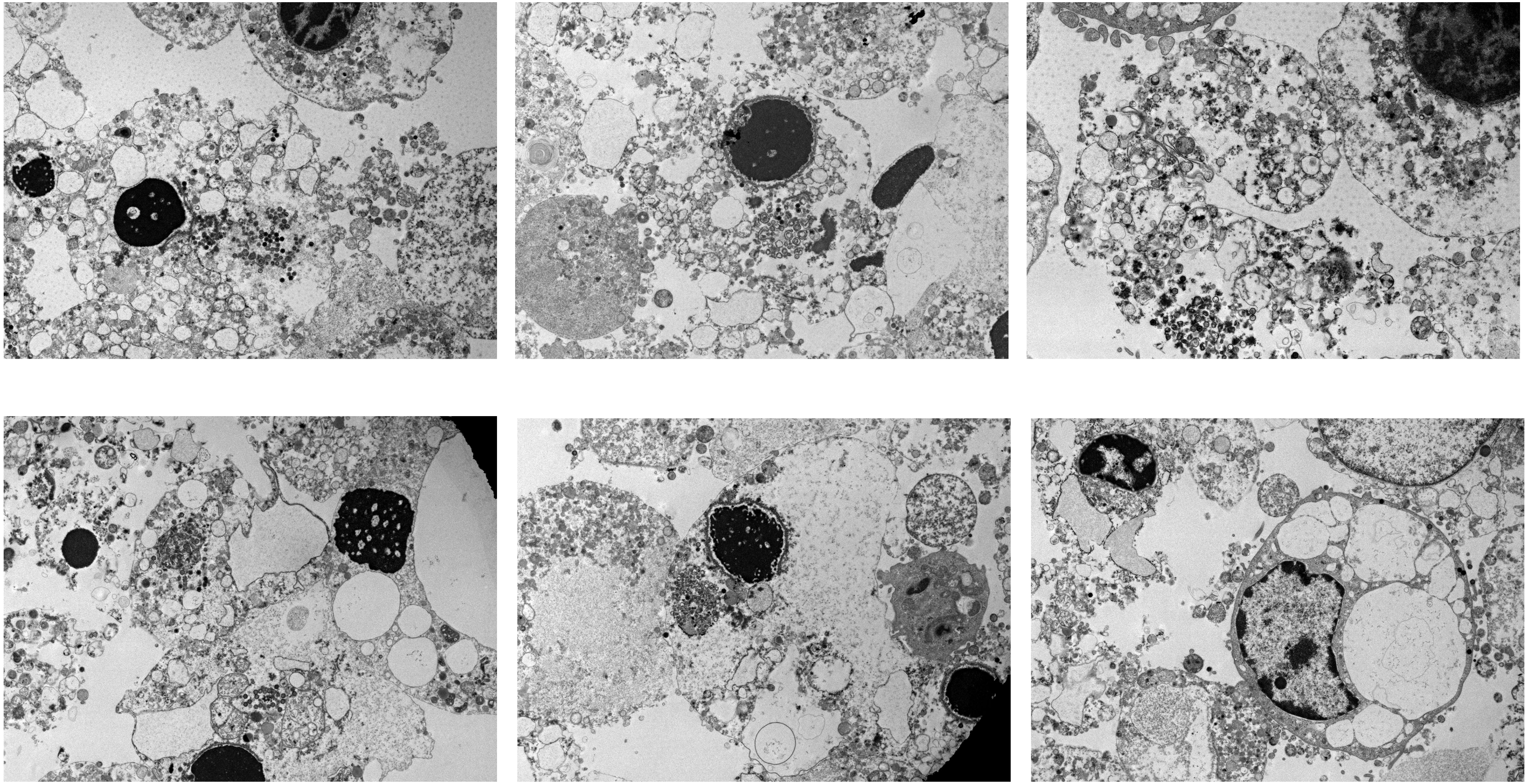

ASFV WT 48 h

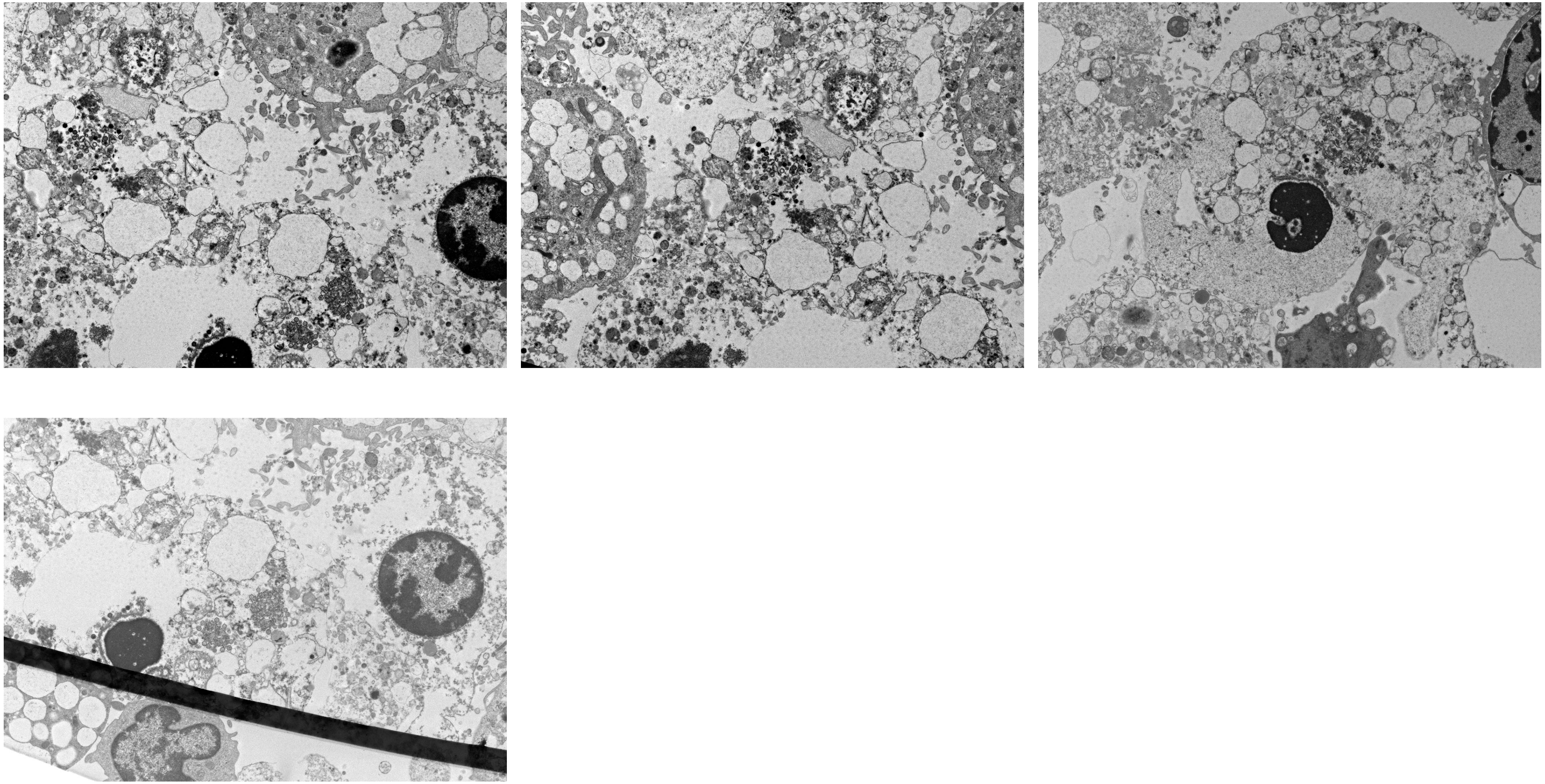

ASFV-ΔE120R 12 h

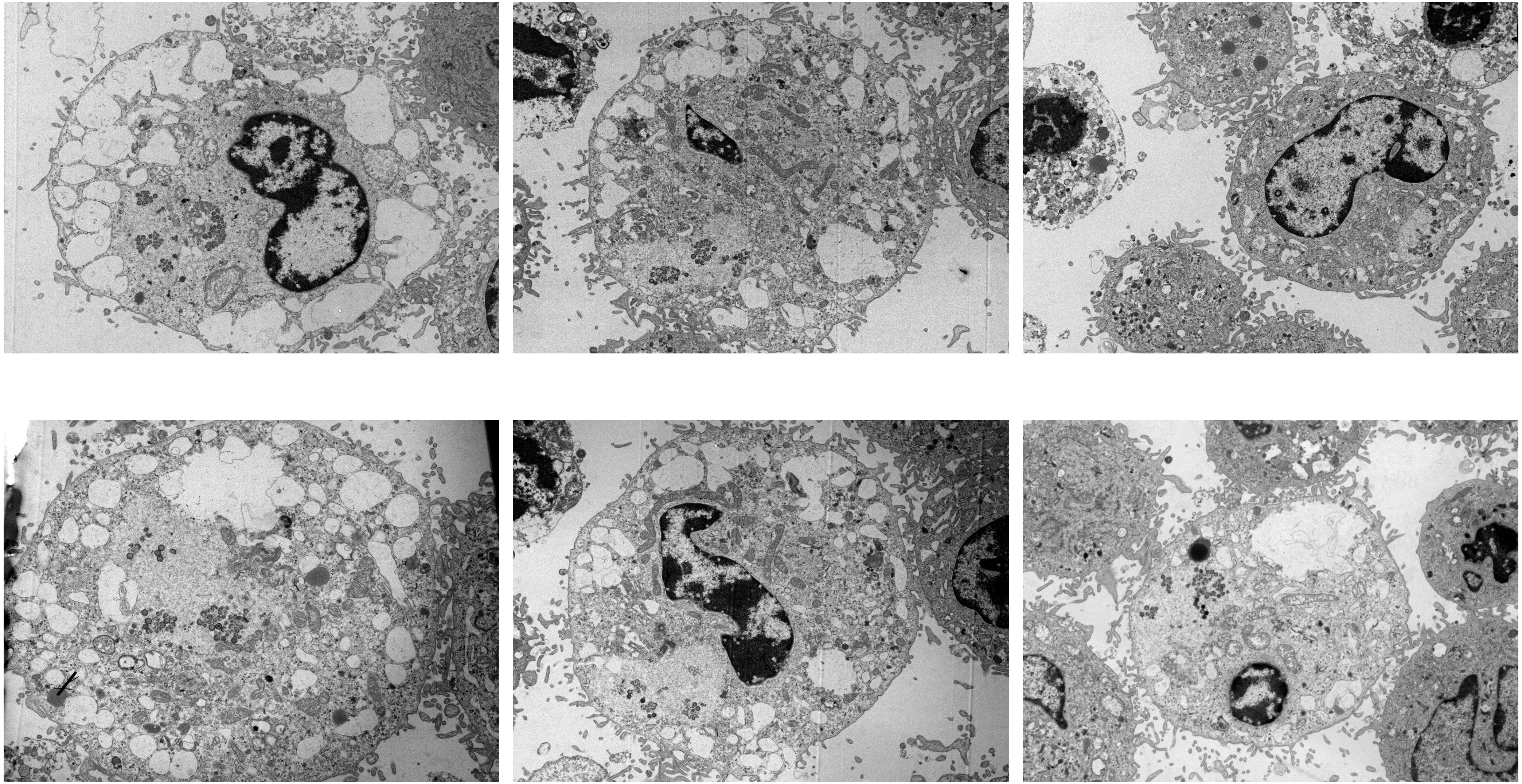

ASFV-ΔE120R 12 h

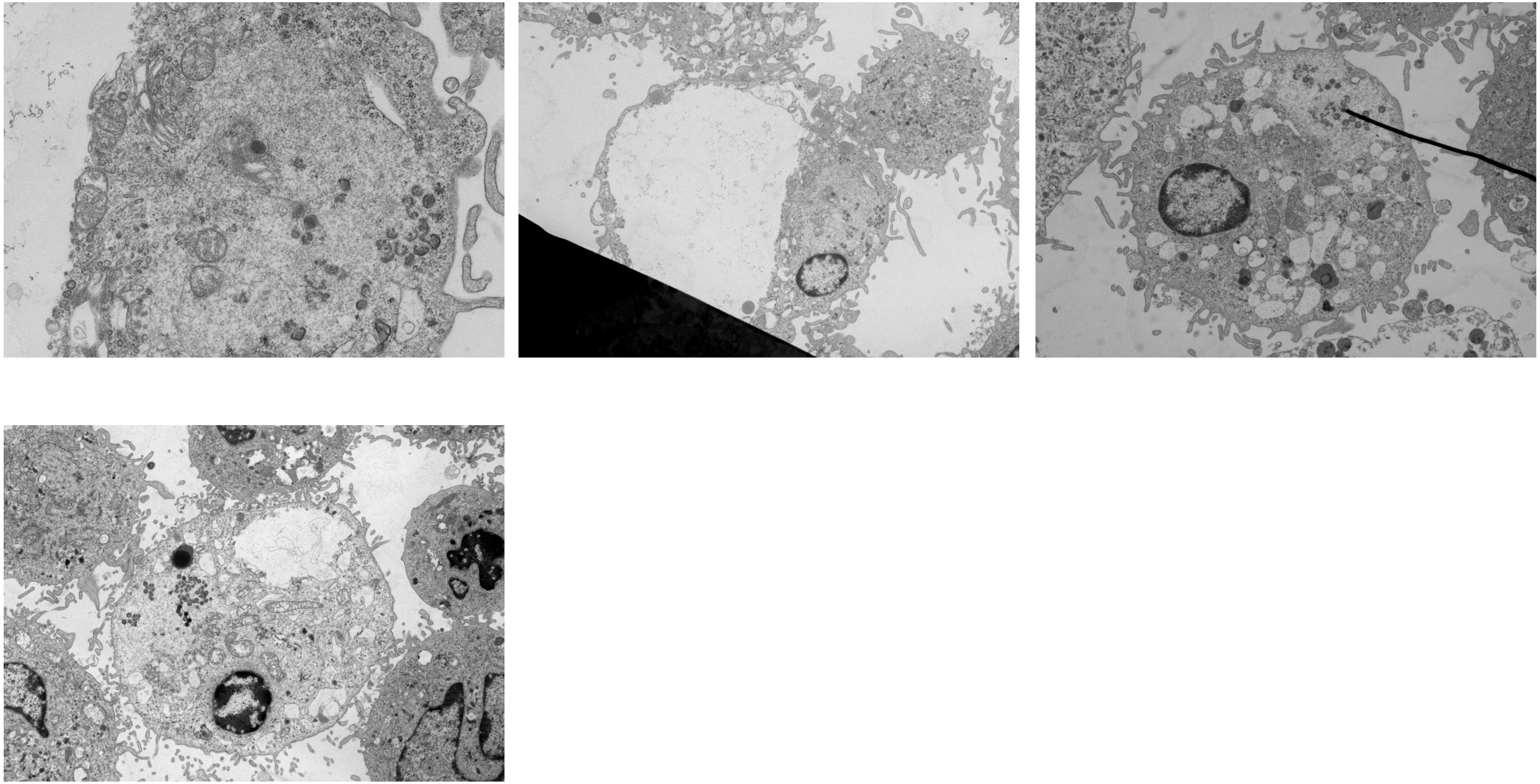

ASFV-ΔE120R 24 h

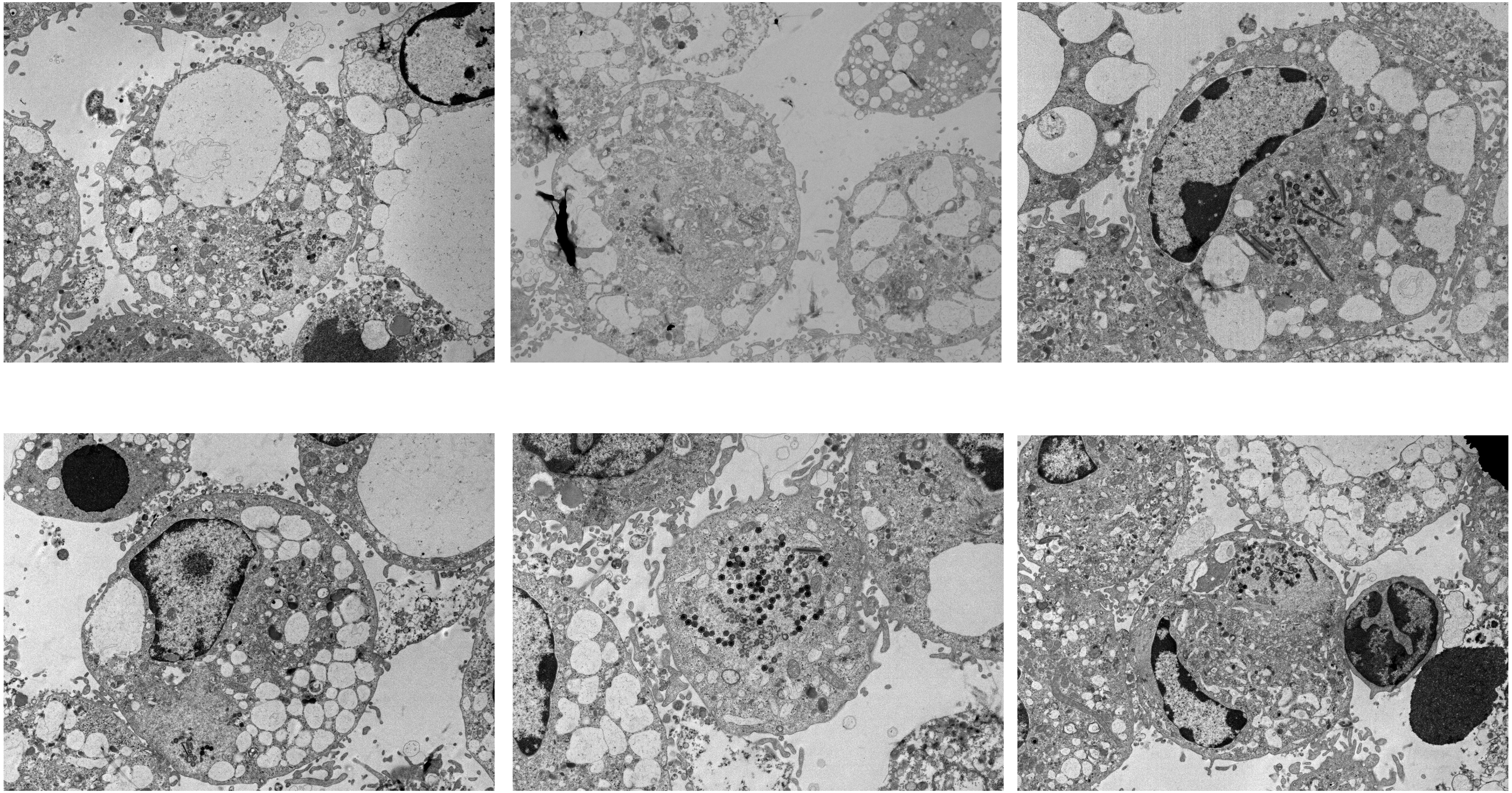

ASFV-ΔE120R 24 h

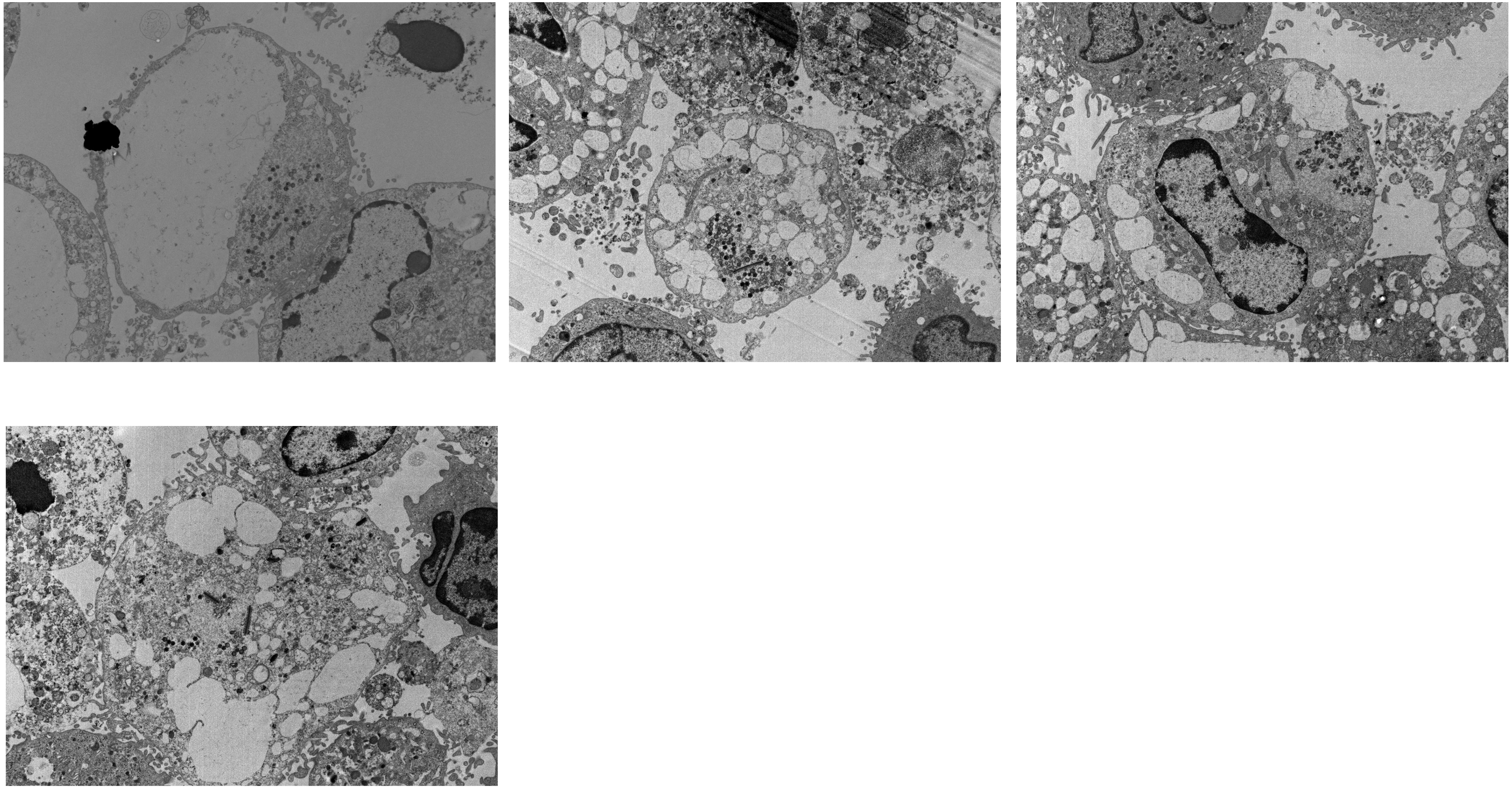

ASFV-ΔE120R 48 h

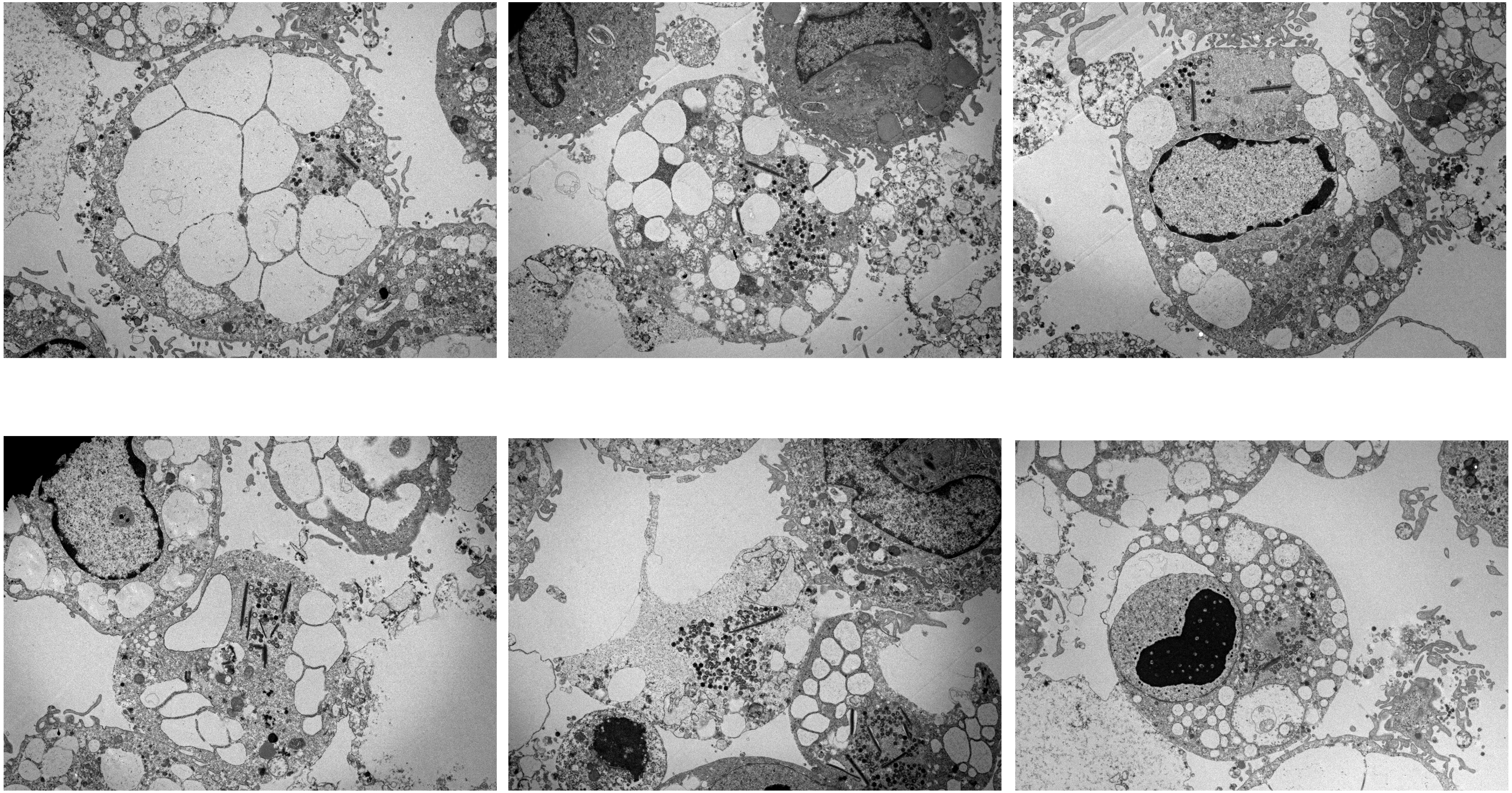

ASFV-ΔE120R 48 h

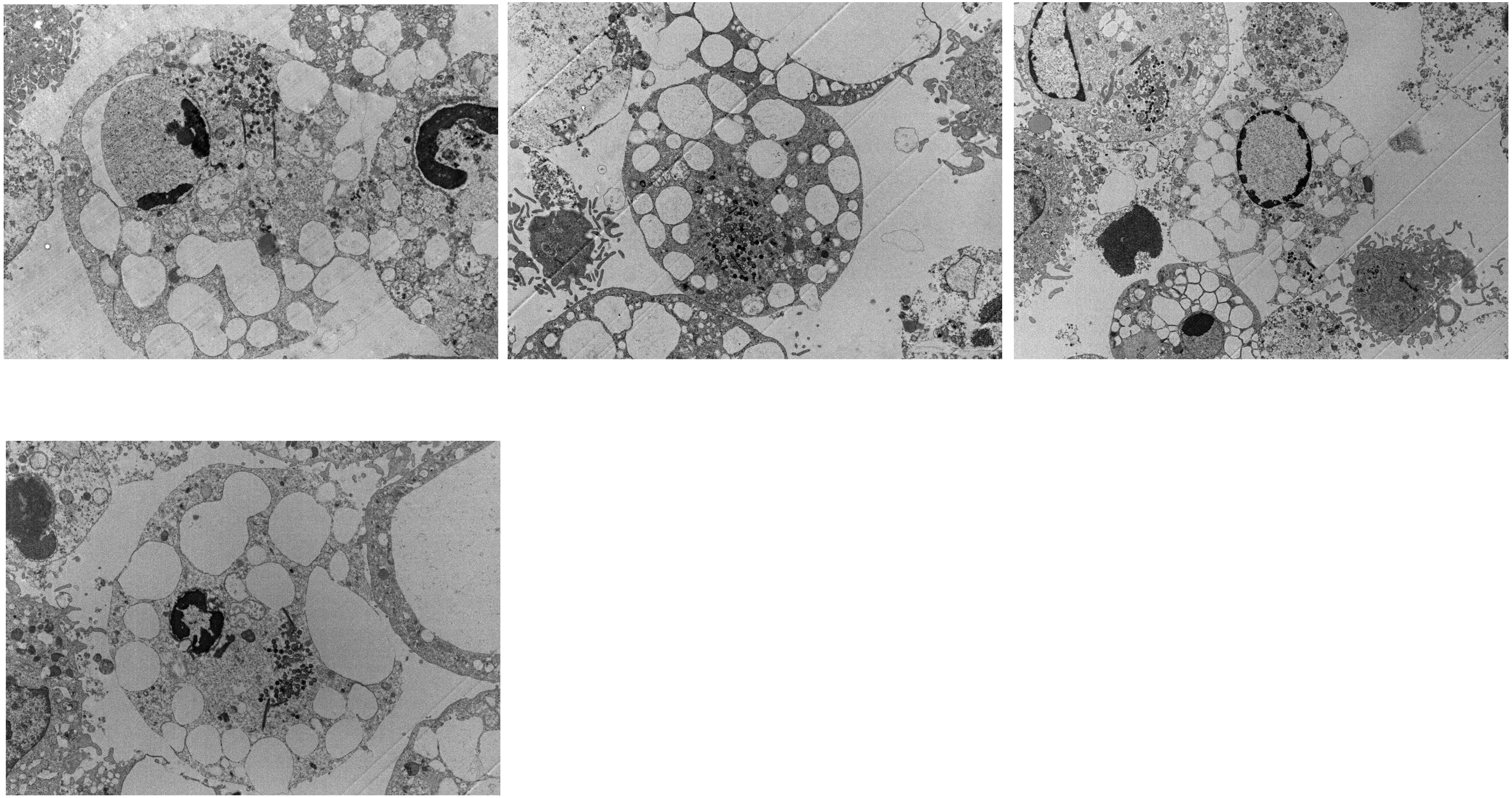

Supplement: Supplementary material_for review.pdf [file TEMI_A_2555722_SM3199.pdf]
